# Part II. Spirit of the Foreign Periodicals

**Published:** 1833-10-01

**Authors:** 


### Spirit of the Foreign Periodicals.

### 1. Remarks on Small-pox, Vaccination, &c.

For the last ten years, the small-pox has been threatening once more to assert its desolating supremacy over the greater part of Europe. In various parts of Germany it had broken out with alarming severity, previous to 1829; and in the Summer of that year it appeared as an epidemic, short-lived indeed, but sufficiently dreadful, in the town of Annaberg and its environs, in the kingdom of Saxony. Such an invasion had not been known there since the year 1800, at which time vaccination was first introduced and generally adopted.

Dr. Otto, who has written a very able and elaborate article on the subject, very justly remarks that it is with vaccination, as with most other objects of man's pursuit and attainment, when once he has reached unto and enjoyed its benefits for some time, its importance becomes less and less valued; and a criminal negligence takes the place of a dutiful and an abiding gratitude. Many families had of late omitted to have their children vaccinated, and perhaps also not a few medical men had not diligently enough enforced the practice, or wisely provided in the performance of the operation for the security of their patients. We do not mean to deny that one year never passed, even when vaccination was most highly appreciated, and most judiciously practised, without some straggling cases of small-pox being known; but we at the same time most firmly maintain, that the two most potent causes of the recent epidemic have been, a certain, although unknown, favouring state of the atmosphere, and a greater and a more generally diffused susceptibility to the disease, in consequence of a deficient and a faulty vaccination. Medical men are still a good deal puzzled what to think of the varioloid disease; and no doubt its appearance within the last ten years has added considerably to the difficulty of arriving at perfectly accurate intelligence on the influence which Jenner's discovery has had on the annual mortality of those countries into which it has been introduced.

Not only those who had been vaccinated, as well as those who had not, but also in many cases persons who had been inoculated, or had passed through the natural small-pox, sickened and died of this new epidemic; still we ought not to forget the very important testimony of Doctors Bell and Mitchell, that its virulence and danger in America were most conspicuously abated in by far the majority of the cases in which the patients had been previously vaccinated, whereas fully one-half of those who had not been vaccinated and had not had the small-pox, died of it.—Truly a mighty service of vaccination! Some physicians, among whom was Moreau de Jonnes of Paris, announced as their opinion, that the varioloid was a disease "sui generis," and that it did not afford any security against an attack of the true small-pox. He considered it as a probable importation from the East Indies.—*(Bullet. des Sciences Med. Dec.* 1826.*)*

Much confusion has arisen from not attending to the form and sort of the disease to which the term varioloid was affixed. Dr. Thomson, of Edinburgh, originally selected it to denote the mild and modified form of small-pox, which occurs in those who have been previously vaccinated; just in the same way as we use the words "alcaloid," "syphiloid," &c. to indicate as minor or less distinct species of alcalies and of syphilis. Tueffard, in his report of the Central Committee of Vaccination in France for 1817, used the appellation "Petite verole mitiguée."

To this meaning the term ought to be restricted; but of late, both in England and in America, and also in Germany, *(Hörn's Archiv.* 1828,*)* physicians have made mention of wide-spread varioloid epidemics; and even the recent invasion of small-pox at Marseilles was by some reported as such!! It has been forgotten that spurious pocky epidemics were well-known, long before the time of Jenner;—*see the writings of Van Swieten, Heberden, Dimsdale, and Hufeland.* We shall there find that the true and spurious forms prevailed epidemically sometimes together; that both left pits and scars behind, but that neither of them afforded any security against an attack of the other. The spurious pox, we are told, runs its course more quickly, the pustules drying on the fourth or fifth day, and the body of the patient does not emit the peculiar small-pox smell. The two diseases are therefore essentially different. Dr. Otto has repeatedly witnessed cases which were mistaken by old and experienced practitioners for examples of genuine variola; and which he at once recognised as spurious, by their shorter duration, and by the absence of the characteristic smell. Two of these patients were seized with small-pox during the recent epidemic at Annaberg, and one of them died. We need not surely urge the great importance of an accurate discrimination. Without it we can never hope to arrive at any safe conclusions. In addition to the two distinguishing signs, it is right to observe that, in cases of varioloid disease, even when severe and when the pustules are confluent, the matter is never so completely formed; that it sooner becomes thick and dry; that the secondary fever is absent altogether, or is very indistinct, and that the pits and scars which remain are not nearly so deep nor so furrowed. As we might expect, there is a considerable difference in these respects, in different epidemics; and perhaps it is generally correct that the severity of the varioloid is in accordance with the malignancy of the co-existing small-pox epidemic. A very satisfactory proof that the varioloid (as previously defined) is only a modification of the true small-pox in consequence of the preceding vaccination, is afforded by the well-established fact that we can inoculate from a varioloid pustule a person who has not been vaccinated, nor yet has passed through the natural disease, and a perfect specimen of genuine variola may follow. It remains for future observers to determine whether the varioloid disease, " as such and retaining all its characters," can propagate itself to, and among those who never have been vaccinated.

The great practical question of the protecting influence of vaccination cannot be fairly disputed. The American and English writers have most forcibly illustrated its efficacy, and Roberts, in his account of the late small-pox epidemic at Marseilles, tells us that 1473 unvaccinated died of the natural disease, and only 45 vaccinated of the varioloid: besides we are to remember that by far the greater number of the vaccinated wholly escaped the infection.

Krausse has calculated that, before Jenner's immortal discovery, 400,000 were annually immolated in Europe by the ruthless monster of variola; and although the number be still very large, and has, no doubt, of late been on the increase, we impute this, not so much to the inefficacy of the antidote proposed, as to the remissness in employing it. Governments ought to view vaccination as a matter of state policy, and to render its adoption exclusive and obligatory. Till then, we cannot hope to effect very much good; for ignorance, indolence, and prejudice are a trio of formidable obstacles. Some of the German states, and among the rest, Saxony, have issued their official mandates in favour of vaccination, but have hitherto omitted to provide for the remuneration of the medical men who were expected to perform it. An adequate fund must be first established, and moreover, no persons should be allowed to vaccinate, except regularly educated and scientific men, whose knowledge may enable them to detect all sources of error, and to correct and obviate them. One of these, which has hitherto not been sufficiently attended to, is the existence of any morbid state, whether of an acute or a chronic nature, at the time of vaccination. From a very long and extensive practice, I

am quite satisfied that the agency of the cow-pox virus may be counteracted, not only by fevers and other pyrexial diseases, but also by a cachectic, scrofulous, or even by a very nervous and irritable state of body. The period of dentition is likewise unfavourable to its success. The vesicle will be found to be imperfectly developed, the crust to fall off more quickly than it ought to do, and the scar left to be neither deep nor indented enough. The age of the child ought also to be well considered: the first three or four months of infant life are occupied in the consolidation of its tender frame, and we therefore observe that, during that period it is very rarely affected by any epidemic or contagious disease. The best writers, as John, Baron, Billiard, &c. have all noticed the little susceptibility of infants to be affected by any of the acute exanthemata. Dr. Otto has never seen any child under four months of age have measles, or scarlatina; and during the late pocky epidemic in 1829, none so young were seized with it.

The observations of Dr. Percival of Manchester, and of Dr. Roberts of Marseilles nearly coincide with the observation of Dr. Otto, as the following tables of two epidemics will shew.

| | |
|---|---|
| Patients under 3 months........ | 4 |
| ——— from 3 to 6 months .... | 17 |
| ——— from 6 to 12 months.... | 119 |
| ——— from 1 to 2 years ...... | 216 |
| ——— from 2 to 3 years ...... | 110 |
| ——— from 3 to 4 years ...... | 59 |
| | 525 |

And again—

| | |
|---|---|
| Patients under 3 months........ | 27 |
| ——— from 3 to 6 months .... | 38 |
| ——— from 6 to 12 months.... | 144 |
| ——— from 1 to 2 years ...... | 200 |
| ——— from 2 to 3 years ...... | 185 |
| ——— from 3 to 4 years ...... | 190 |
| | 784 |

The occasional occurrence of small-pox during fœtal life, and also from the birth is a curious fact; but it does not at all invalidate our position, that infants are very little susceptible of the infection.

The alarms occasioned by the recent increase of small-pox, and the consequent discredit which has to a considerable degree been thrown upon vaccination, has induced several practitioners to recommend the operation to be repeated after the lapse of a certain number of years, not only with the view of additionally fortifying the constitution against the variolous poison, but also at the same time of testing the sufficiency of the first vaccination. It had been supposed that a tolerably correct opinion might be formed by examining the scar on the arm; but this has been found to be wholly erroneous. Dr. Otto has repeatedly had cases of severe and even fatal small-pox in those whose arms presented a most distinct and well-marked cicatrix, while others in whom the traces were little obvious, have wholly escaped. As to the question, whether the protecting influence of the cow-pox remains only for a period of years, and not for the life of the individual, and whether it requires therefore an occasional renewal, we cannot, in the present state of our knowledge, accurately determine. It seems more probable that the influence of the unfavourable agents, which we have previously alluded to, namely, unfitness of age, existing diseases, or a diseased constitution, dentition, and so forth, operating at the time of the vaccination, may have thwarted the full efficacy of the antidote.

We must therefore satisfy ourselves with carefully collecting and comparing facts; and with this view we shall now lay before the reader the results of 189 "re-vaccinations" performed in the year 1829.

In 76 cases, papulæ, or inflamed elevated points only and without any appearance of a pustule or of a vesicle, were induced. Although there was no obvious exudation, a small crust was formed on the top of these; it fell off on the 6th or 7th day, and left no scar, nor any trace behind. In these cases, I consider that the influence of the original vaccination was in full force, and not at all impaired.

In 83 cases vesicles were formed; these were usually filled with a turbid, thick matter, on or about the fifth day; on the seventh or eighth day, they had become dry, and on the tenth, the scab fell off, leaving a very imperfect mark. Such an eruption bears a considerable resemblance to varicella, and as I regard it as a false or spurious cow-pox, I shall call it "vaccinella."

In nine of the cases papular elevations appeared about the third day; and like regular vaccine vesicles, were gradually filled with a serum, but dried and passed away more quickly, and without the formation of any purulent matter. More or less feverish disturbance accompanied their progress. On the falling off of the brown-coloured scab on the 10th or 11th day, a superficial scar on a red ground, remained. This eruption may therefore be viewed as a modified cow-pox, and as probably indicating the disposition of the individual to the varioloid disease.

In sixteen cases, perfect, and in all respects regular and well-formed cow-pox vesicles were formed, and were followed by the true pitted scar. In five other cases the vesicles were nearly as satisfactory. The conclusions which may legitimately be drawn from the foregoing statements are, that out of the 189 persons who underwent vaccination a second time, 76 may be considered as retaining completely, and in a full degree, the protecting influence of the cow-pox; 83 in a high degree; 9 in a doubtful and uncertain degree, and 21 as having lost it altogether. Of these 21, ten who had been all vaccinated in infancy, were between 10 and 15 years of age; 7 between 15 and 20; and 4 between 20 and 27 years. Some of these had been vaccinated at different periods of their youth, in their first, second, or third years. Hence it appears, that the smallest number is actually of those in whom the first vaccination was least recent. How shall we explain this? Is it owing to a better mode of vaccination, or a more efficient virus then employed? or is it to be attributed to a diminished susceptibility to the variolous disease, as we advance in years? As we before stated, the appearance of the old scar is quite fallacious, since it was well-marked in some of these 21 cases, and it was very indistinct in many of those who resisted the effects of re-vaccination.

Numerous cases of small-pox which occurred during the late epidemic at Annaberg, are detailed by Dr. Otto, but we deem it unnecessary to transcribe any of them.—*Hufeland's Journal, March.*

---

### II. ON THE INFLUENZA AT BERLIN.

Dr. Hufeland, in the March Number of his Journal, alludes to the then prevailing epidemic influenza, or grippa, which subsequently, as all our home readers well know, extended itself to this country, and spread like a broad sheet over almost every hole and corner of it. The venerable German tells us that, since the year 1782, no epidemic has been known to seize so many persons; in many places, more than one-half of the inhabitants were affected with it. Both arose in Russia, and followed a south-westerly direction; both made a sudden invasion on a vast number of people in a place at the same time; both were of short duration, and were comparatively little dangerous; both affected chiefly the mucous membranes and nervous system; and in both bloodletting and depletory measures were hurtful. In Petersburg, there were at least 100,000 invalids; in Memel, whose population does not exceed 10,000, there were 8,000; and in Berlin, at the date of Hufeland's writing, upwards of 50,000 had been seized. It may be considered as a catarrhal fever, accompanied with, and followed by, extraordinary depression of the nervous energy for several weeks after the pyrexia has ceased. Mild antiphlogistic treatment and gentle diaphoretics have, in most cases, been sufficient to cure it, even in this "*blood-thirsty age*" of ours.

A correspondent from Konigsberg adds a few interesting remarks on the epidemic, as witnessed by him there. The Winter had been unusually healthy up to the end of the first, or the beginning of the second week in March. The writer, as well as some other physicians, had, indeed, remarked that there had been, for some time previous, a tendency in most febrile complaints to a nervous or adynamic type; and this is quite in accordance with the history of other epidemics, as, for example, of the cholera: the influence of the stormy cloud is felt, before it breaks in its full sweeping force. The symptoms were at first smart pyrexia, with very severe headache, sneezing, sore throat, and violent cough, which was generally dry and harsh, at least in the beginning; the skin moist, and the tongue white. The feelings of general pain, weakness, and great depression of nervous power, were very remarkable. The fever generally abated in three or four days, but the patients were long of recovering their strength. The mortality occasioned by the disease was very trifling, if we consider the number of patients, and occurred chiefly among children, in whom bronchitis was developed. Almost every one in Konigsberg was affected with it, in a greater or less degree; some, indeed, very mildly, but still they had catarrhal symptoms. During its prevalence, other diseases were arrested, and seemed to slumber for the time; the sick lists presented nothing but influenza—influenza!! Commerce was frequently suspended, and churches had no clergymen to officiate in them. In the course of the second week, the disease became less severe; in some, the fever was absent—in others the headache, or the sore throat, or the cough, and so forth; but such patients were often much longer indisposed than those who had sustained a smarter attack during the first week; whether this arose from their taking less care of themselves, or whether the "potentia nociva" required a certain time for its maturation in, and expulsion from, the system, we cannot say. In the third week, the number of cases was very much diminished, and so disarmed now was the disease of its violence, as to receive the appellation of "grippine," a diminutive of "grippe;" in the fourth week, scarcely any new cases were seen. The mortality may be estimated by the following table:—We should premise that the average weekly mortality at Konigsberg is from 40 to 50 in Summer, and from 50 to 60 in Winter.

| | |
|---|---|
| Deaths from 8th to 15th March.. | 43 |
| ——— from 15th to 22d ........ | 72 |
| ——— from 22d to 29th........ | 105 |

The last is a greater number than has been known for many years, except when the cholera was raging. During the epidemic influenza of 1831, the highest number of deaths in a week was 96.—*Hufeland's Journal.*

### III. Coincidence of Human Epidemics with those among Fishes.

In 1784, several of the Indian tribes in the state of Massachusetts suffered very severely from a pestilential disease; and it was remarked that, during its prevalence, all the "blue-fish" had disappeared from the sea on that part of the coast. What is singular is, that since that period they have never returned in any numbers; and previously so abundant were they, that the fishing was a lucrative employment.

Every one has heard of the extraordinary mortality, in the year 1789, among the haddocks off the shores of Norway, Scotland, &c. The journals of the day give ample accounts of it. Captain Steward, who commanded a vessel from Archangel, tells us that the sea was covered, for several leagues in extent, with the floating dead fish. Now during this time, several parts of Scotland were ravaged with a most malignant scarlatina and cynanche. The reader will find some very interesting details in Sinclair's Statistical Reports. One of the most memorable years, for the coincidence of several of Nature's dismal disasters, was 1756. Lisbon was rent to pieces; the island of Mytilene, in the Archipelago, was shaken to its centre; North and South America felt the heavings of a general earthquake, and Austo was literally annihilated. Ætna and Hecla were lighted up with double fury, and England, France, and other countries were ravaged by a formidable epidemic of putrid sore-throat, and Constantinople and the East by a destructive plague. The same year was remarkable for the vast numbers of whales and other fish found dead on various parts of the ocean. Again, in 1775, the state of Connecticut, in North America, was visited by a very fatal dysentery and by a cynanche maligna; and the waters of the harbour of Welflect, near Cape Cod, were so affected, that all the oysters died—*vide* Dr. Webster's interesting work. A similar mortality among oysters has been known to accompany severe epidemics of the yellow fever, as in the years 1793, 4, and 6. The old author Cedrenus relates that, during the dreadful pestilence which brooded over almost the whole earth, in the latter part of the sixth century, a vast quantity of fish died in many places; and we read in the Universal History of Magdeburg, and in the tenth book of Baronius, that "a pestilence which was truly most fatal to the human species, was no less so to aquatic animals; for the banks of rivers were covered with dead fish, which putrefied and infected the air with an intolerable stench." Nicephorus and Echard mention that there was a terrible mortality among whales, about the middle of the fifth century, when the Roman Empire, under the reign of Theodosius, was desolated by a plague. It was believed that these monsters of the deep had been battling with each other; but such glorious results of war appertain to man alone!!—*Alibert in Revue Medicale.*

---

### IV. Large Doses of Antimony in Phlebitis and Pulmonary Hæmorrhagy.

An interesting paper on the therapeutic properties of antimonial medicines, given in large doses, appears in one of the May Numbers of the Journal Hebdomadaire. Few, in the present day, will dispute their strikingly beneficial effects in acute pneumonia. The testimony of Rasori, Laennec, Recamier, and Ribes leaves no room for scepticism, although the modus operandi is still a question of discussion. We are told by some that they are equally efficacious in arresting the progress of phlebitis; a drachm and a half of the white oxyde may be given repeatedly. M. Sanson has frequently employed it, and also the emetic tartar, in the dangerous inflammation of the veins which follows surgical operations. MM. Trousseau and Bonnet, the authors of this paper, have successfully exhibited them in a case of phlebitis, where the inflammation had seized both of the lower extremities of a young female, six days after her confinement; and also in two cases of puerperal metro-peritonitis.

Professor Recamier has treated the parenchymatous hæmorrhage of the lungs by antimonial medicines, with very decided benefit. A young woman had suffered from this malady for ten months, and had been bled 29 times. A variety of remedies had been ineffectually tried; but on giving large doses of the tartrite of antimony, she was speedily and permanently cured. A man, aged 40, was brought to the Hôtel Dieu on the seventh day of an alarming hæmoptysis, which had rather increased than abated after bleeding. Antimony was given, and in seven hours the bloody expectoration had ceased. The same treatment succeeded admirably in the case of an old woman, who had suffered repeatedly from attacks of pulmonary apoplexy, symptomatic of diseased heart. It is not, however, to be denied that, in other cases, we have failed.

In bronchial hæmorrhage, the effects of the remedy have not been at all encouraging.

In pleurisy, it has in our hands utterly failed; and in acute rheumatism it is of very doubtful utility. MM. Dance and Chomel contend that it is beneficial only when, and in proportion as, it vomits and purges; and this opinion seems to have been held by the older practitioners, for antimony is by no means a new remedy in rheumatism. There is much wholesome and sensible observation in the following extract from old Salmon's Praxis Medica, London, 1707:—"The first thing to be done is to give a vomit of tartar-emetic, and in three or four days' time to repeat it; which being given a third time in some hundreds, to my knowledge, has absolutely done the cure without any other means. But if the disease be rebellious, and will not submit to this, you may, after the vomits, give two or three purges of the pilulæ mirabiles, or those of Chalmatæus, which seem to take away the pains as if by enchantment, as these pills mostly do; yet, in some patients we have met withal, they have been ineffectual. In this case, we have been forced to have recourse to the aurum vitæ, or turpeth mineral, which may be given in doses of from two to four grains, for two nights together, intermitting two nights, and then repeating the doses for two nights more; and thus continuing for six or eight doses, till a gentle flux does arise, which does truly, and beyond all other means, most effectually cure this disease. I speak from a long and thorough-tried experience. But in some persons, where I would not have a salivation or flux, I have given gentle and pleasing cathartics, in the intervals of the two and two doses; and these methods I have never found to fail."

---

### V. Variola and Varioloid.

In a late report of the clinique of the La Pitié Hospital, we observe that several severe cases of natural small-pox are detailed. They occurred in unvaccinated persons; and were fatal in two. In one of these, a copious eruption was found on dissection, over the engorged lining membrane of the air-passages, and several ounces of a thickish purulent matter flowed from them; the patient died asphyxiated; the right lung presented here and there patches of hepatisation. In the other case, a disseminated suppuration in the pulmonary parenchyma was found after death, which had taken place on the 28th day of the disease.

Two cases of the varioloid were admitted;—in one, a female, aged 22, who had been vaccinated when young, but in whom the scars on the arm were no longer visible, the disease ran its course in eight days: in the other, a man aged 28, and never vaccinated, the eruption appeared on the third day, and terminated favourably in a week. What is interesting is, that this patient seemed to have caught the disease while tending a younger brother sick of the regular small-pox; and yet we cannot hesitate to pronounce his own attack as one of the varioloid, when we consider its duration and progress. At the Beaujon hospital we lately took notice of a patient who was affected with varioloid, and yet gave distinct and well-marked small-pox to several others in the same ward.—*Archives Générales.*

---

### VI. Paraplegia in a young Female, cured by Nux Vomica.

A girl, aged 20, was admitted into La Pitié on the 13th January, for a paraplegic weakness of the lower extremities; she had ready command over the muscles; but their energies were so feeble, that she could not walk, nor even stand erect, but for a few minutes; the toes were in a constant state of extension; and upon any attempt to advance, the thighs bended upon the pelvis, the gait became unsteady and tottering, the feet crossed and became entwined with each other, and she would fall on the ground if not supported. This loss of power was most marked towards evening, and also during the periods of menstruation. The sensibility of the limbs was unaffected, and her constitution sound in other respects. The disease had commenced in her 11th year.

The alcoholic extract of nux vomica was administered daily in an enema; the dose at first was two grains, and gradually raised to five. On the fourth day, the power over the limbs was somewhat greater, and the catamenia were induced. Latterly the strychnine was given by the mouth in the form of pills in doses of $\frac{1}{8}$, $\frac{1}{4}$, $\frac{1}{2}$, and 2-3ds of a grain. In two months and a half she was discharged cured.—*Ibid.*

---

### VII. Frequency of Calculous Disorders in Egypt.

Medical writers have frequently committed the error of asserting that calculous diseases are unfrequent in warm climates. The statement is perhaps true as a general, but not as a universal rule. In Egypt they are of common occurrence, as appears from the report of M. Clot Bey, who has operated forty times for the stone, since his residence in that country. Prosper Alpinus, in his treatise on the Diseases of the Egyptians, alludes to the frequency of urinary calculi; and attributes the predisposition to the irritation and weakness of the kidneys, induced by excess in venereal pleasures, and the more immediate cause of their formation to the sand in the waters which they drink. Most indeed of the patients are from among the inhabitants of Lower Egypt; a few, however, are from the more central division of the country. M. Clot has never seen a case among the Nubians or Abyssinians. The cause of this inequality, he thinks, may be found in the humidity of the air and the unwholesomeness of the water in Lower Egypt. The ground lies very low, and is frequently covered with stagnant muddy water, which is used by the lower orders as drink; the better classes have it previously filtered, and are thus more exempt from its injurious effects.

Egypt, like other countries, is infested with swarms of empirics who play on the credulity of the vulgar, by boasting of their infallible solvents for the stone. The people being ignorant and superstitious are easily duped by these charlatans, for every affection of the urinary organs is by them set down and treated as stone in the bladder; and no doubt many of the supposed cases (they never use a sound) get well under their treatment. There is another method sometimes employed by the Arabs, and that is, the forcible blowing of air into the bladder, and then sucking it out, while the hypogastrium is at the same time strongly compressed; the cunning dogs know full well that no stone can come out, and therefore conceal one in their mouths before they commence the suction; the patient is satisfied when he sees *a*, if not *the*, stone, and gratefully rewards the imposter. Lithotomy has however been long known and practised by the Egyptian surgeons; there are two methods in use; one is the perineo-vesicle, or nearly the Celsian, the other is the recto-vesical.

In both, two fingers of the left hand are carried deep into the rectum, in order to grasp and confine the stone, and to make it protrude as much as possible; a deep incision is then made directly upon it, and the fingers of the right hand are generally used as forceps to withdraw it. It must be acknowledged that very few patients die of the operation, although most of them afterwards labour under urinary fistulæ, or incontinence of urine.

The recto-vesical method is generally adopted; it is at once easy of execution; a large stone may be most conveniently withdrawn; and the risk of hæmorrhage is less than in any other. Many of the native lithotomists acquire great adroitness; they practise no other part of surgery; and the profession or trade is handed down from father to son, often for many generations.

The following is a translation of an old Arabic work, written eight centuries at least ago. "When you intend to extract a stone from the bladder, you should order a person to seize the patient under the shoulders, and then lift him up several times, shaking him heartily so that the stone may fall to the lowest part of the bladder. The patient should also be instructed to leap from a height, or to dance. He must then lie down, with his legs bent upon his thighs, and his hands secured below his knees, in order that the bladder may be protruded downwards. The operator is now to press forcibly upon the hypogastrium with the one hand, and with the other to examine the perineum if the stone can be felt. If he feels it, let him cut at once upon it; if he does not, one or two fingers are to be introduced into the rectum, to bring down the stone to the neck of the bladder, pushing it out to make it project; the assistant is now to keep the testicles up, and the surgeon is to cut between them and the anus, inclining the incision outwards to the left hip in an oblique direction, in order that the opening be large and proportioned to the size of the stone. If the finger in the anus be still kept pressing upon the stone, it will probably now leap out of itself; but if it does not, then an instrument is to be employed to extract it. The operation being finished, some yellow powder is to be sprinkled on the wound, a compress is to be applied to it, and then a bandage, which is called a 'bride.' The patient must lie on his back, and endeavour to make water whenever he has the desire, in order that it may not accumulate in the bladder, for this would retard the healing. He ought to wet the wound frequently with a lotion of vinegar and rose water. On the third day the bandage is to be removed, and the wound to be dressed with black ointment. If the parts become inflamed and swollen, they should be anointed with appropriate salves, and the aperture is to be bathed and injected with an infusion of chamomile, or with melted butter, and if God wills, the patient will recover."

Out of the thirty-eight cases of lithotomy performed by M. Clot, eleven were cured from the 7th to the 10th day after the operation; sixteen from the 11th to the 20th; eight from the 22d to the 30th; four from the 32d to the 40th; and one from the 40th to the 50th day. He has lost only two patients, and three were discharged with vesico-rectal fistulæ.

The operator very modestly ascribes his great success to the fine climate of Egypt, which is favourable, he says, to the healing of all wounds; and also to the temperament and constitution of the people being little irritable and not easily excited. This remark had been previously made by many of the surgeons of the French expedition, and especially by Baron Larrey. In five of his cases M. Clot performed the recto-vesical operation; in three of these fistulæ remained uncured. He admits that the operation is exceedingly easy of execution, and that very large calculi may be conveniently extracted; but he has abandoned it for the rapheo-vesical method proposed by Vacca, and which he has performed eleven times; the stone is extracted at the most roomy part of the perineum; no important blood-vessel is exposed to the knife, and the rectum can with difficulty be wounded. The only serious objection which has been urged against it, is the danger of wounding the seminal tubes; but we should remember that they may be wounded in some of the other operations, and moreover that only one of them can be divided, the other remaining safe and perfect. Besides, may not a vas deferens, like any other tube, unite, after being cut across?—*Annales de la Medecine.*

---

### VIII. On the Rhinoplastic Operation.

M. Labat, ex-surgeon of the Viceroy of Egypt, has contributed a humorous paper on the subject of Rhinoplastie, or the art of nose-making, to Broussais' Annales. We shall make a few extracts for the amusement of our readers. No deformity, we are told, is more disgusting than the loss of a nose. History informs us that the chaste Eusebia, abbess of the Monastery of St. Cyr, at Marseilles, and 40 of her nuns, cut their noses off, to save themselves from being ravished by the Saracens, who had gained possession of the town—a dreadful alternative! The English chroniclers relate instances of equal magnanimity on the part of some of their fair countrywomen, during the period of the Danish invasion. From Diodorus Siculus we learn that, by command of Actasan, the noses of all the inhabitants of Kistipoor were cut off, in order that these robbers and assassins might be known wherever they went; their town was afterwards called Nasica-Tipoor. Among the Egyptians, Greeks, and Romans, the loss of the nose was the punishment inflicted on adulterers; and, to add to ignominy, the injured husband had the privilege of being the operator. No doubt the incisions were, according to the precepts of modern surgery, free and extensive! M. Labat was travelling through Egypt in 1824, and arriving at Foha, he observed a great number of people who had lost their noses. His guide informed him that the neighbouring district had long been infested with bandittis, and that it was necessary to inflict a severe punishment upon such robbers as were seized, in order to intimidate the rest. The same penalty, was awarded, also, to adultery, that crime being considered as the arch or master-theft of all. Our readers may recollect the lines of the poet Musianus—

Si mæchis rasum mos esset tollere nasum
Multi per mundum sine naribus esset eundum."

The English Queen Elizabeth, noted for her large nose, ordered that all who spoke ill of her person or of her government should have their ears and noses cut off. Charles the 2d punished one of his noblemen, for some satirical observations, by the loss of his nose; and who has not heard of the notary's wife in Paris, who wreaked her vengeance on her husband's paramour, by tearing her nose clean away!

There are many curious details in the traditional history of the nose. According to Herodotus, it signified, in the symbolical language of the Egyptian ritual, a wise and cautious man. We know how highly the Greeks and Romans esteemed the long and square-pointed nose—"longus, quadratusque nasus," and many of their leading citizens thus derived their appellatives, as Ovidius Naso, and Scipio Nasica. The French language has borrowed many idioms from this most honourable member—thus, we say "*il a un bon nez,*" to mean a discreet person; "*on lui a donné sur le nez,*" a mortified person; "*se casser le nez,*" an unsuccessful person; "aire au nez," to rail or ridicule another; and we even apply the terms "nasilard, nasiloques, homme qui parle du nez," to the poor unfortunates who are noseless altogether, following, no doubt, the practice of the scholiasts, who derive their "lucus a non lucendo." Travellers assure us that the Tartars squeeze and flatten their children's noses, for the sapient reason, that it is a great folly to have one's nose sticking out in the way of the eyes. Their neighbours the Brahmins, on the other hand, seem to have been the original nose-manufacturers. Galen travelled in the East, and gives us a description of the operation; the skin of the cheeks was drawn forwards, and, to facilitate the extension of the integuments, several longitudinal incisions were made in them, so that the flaps might be brought into contact with each other from either side.

Celsus recommends that the incisions be made close to the ears; and in other respects he follows the preceding method—"non creatur ibi corpus, sed ex vicino adduntur; quod etsi in levi mutilatione fallere oculum potest, in magnâ non potest." Baron Larrey has successfully employed the ancient operation in two cases—*vide* Journ. Complem. des Sciences Med. Mai, 1821, and the the 4th vol. of his works. The plan proposed by Dieffenbach, differs little from the rhinoraphy recommended by Celsus. That curious old boy Olaus Magnus, suggests the use of a piece of the flesh from a living fowl, as a substitute for the lost nose!! In the 16th century, two brothers of the name of Boiani, Neapolitans, acquired prodigious fame by their skill in repairing noses; unfortunately for poor Tycho Brahe, who lost his nose by a sabre-wound in a duel, they did not visit Denmark. In 1587, Tagliacozzi published a tract, entitled "Epistola ad Hieronimum Mercurialem de Naribus multo ante abscissis reficiendis;" and ten years after his "Chirurgia Nova de Narium, Aurium, Labiorumque defectu, per Insitionem Cutis ex humero." So proud were his fellow-citizens of Bologna of this celebrated rhinoplast, that they raised a statue to his memory, and in his hand he held a nose! The taliacotian cure was a very tedious and fatiguing one to the patient; for, as the flap which was to serve for the new nose was obtained from the arm or fore-arm, it was necessary that the upper extremity should be kept folded across the face, so that the bleeding wound on it, and the raw edges of the nose, be kept accurately in contact, until perfect union had taken place; for not till then, a period usually of 30 days, was the radicle of the flap to be divided. Tagliacozzi particularly recommends that the nose be made large enough—" melius est amplas gestare nares, quam imminutas et deformes." a modification of the above operation was tried by some surgeons. They made an incision through the integuments of the middle and anterior surface of the fore-arm; they then made the edges of the mutilated nose raw, and applied them to this wound, retaining them in accurate contact with each other. At the end of 15 or 20 days, when adhesion had taken place, two triangular flaps were dissected from the adjacent integuments of the fore-arm, and left hanging to the nose; these were then brought in apposition with each other, forming thus a raphé, or bridge of the nose, and retained by sutures.

Graeff of Berlin, and the late M. Delpech of Montpelier, are the only modern surgeons who have practised the old Taliacotion method of taking the flap from the arm; they have each indeed devised particular apparatuses for the purpose of confining and retaining the arm to the head; but all the essentials of their operations coincide. In Graeff's first case it was little short of a twelvemonth before the new nose was fairly completed; and then it was such a very curious nose, that the patient exhibited himself for money to the wondering eyes of his compatriots. The details are communicated in Hufeland's Journal and in Baron Percy's work. His second case was that of a young woman, who had lost her nose from a phagedenic ulcer. The flap was dissected from the inner side of the arm, but left attached by its base; the raw edges of the nose and of this triangular flap were then united by means of needles and sutures, the arm being necessarily secured to prevent all movements. Adhesion had so far taken place by the end of the fourth day, that Graeff removed the sutures; and in two days more, he detached the base of the flap from the arm; on the twenty-sixth he paired and fashioned it into a becoming shape, the apertures of the nostrils having been previously made and kept open by cylinders of cotton; and the cure was completed in a fortnight afterwards.

[*To be continued.*]

---

### IX. Gastrotomy, in a Case of Extra-Uterine Pregnancy.

A negress at Pernambuco in Brasil consulted Dr. Benit in May 1830; she expected every day to be delivered; labour however never came on regularly, and soon all pains left her entirely. The swelling of the abdomen was as great as ever; and in course of time, the poor woman's health began to decline. In May 1832, an abscess formed about the navel, and some fœtal bones were discharged. Dr. B. then determined to lay open the abdominal cavity by an incision of three or four inches in length, and using his fingers as forceps he extracted the putrid remains of a decayed fœtus; the stench was most offensive. The edges of the wound were brought into contact, and the patient confined to a rigorous antiphlogistic treatment for two months. Ultimately she quite regained her health.—*Annales de la Med.*

---

### X. Vaccination.

Professor Lobstein, of Strasbourg, has lately published an interesting history of the variolous epidemic, which prevailed during the year 1825 and 26 in the department of the Lower Rhine. At the present moment, when the stability of Jenner's great discovery seems to be doubted by many, it cannot fail to be instructive to know the sentiments of such an able observer as our author. The inferences he has drawn may be reduced to the four following.

1. That by far the greater number of those who sickened during the epidemics were unvaccinated, or those in whom vaccination had not succeeded.

2. That when the disease attacked those who had been successfully vaccinated, it was invariably mild, and not unlike to varicella.

3. That the striking difference which was universally observed in the severity and fatality of the disease, when it occurred in unvaccinated, and when in vaccinated persons, can be attributable solely to the protecting influence of the cow-pox.

4. That therefore the cow-pox must be still considered as a most valuable, although not an infallible preservative against small-pox, and that when small-pox does supervene, it is usually mild and much modified.—*Archiv. Gener.*

---

### XI. ÆTIOLOGY AND TREATMENT OF GANGRÆNA SENILIS. By DUPUYTREN.

A man, aged 71, was admitted last March into the Hôtel Dieu. For several days he had suffered from pain and a sense of coldness in the left great toe; it was swollen and rather livid; the pain got worse; a phlyctæna formed, broke, and discharged a brown-coloured fluid; and the subjacent parts were found to be mortified; the eschar extended to the meta-tarso-phalangeal articulation; it was hard and dry; the pain was now so severe as to prevent all sleep, and to induce feverish symptoms. The heart and great central vessels were ascertained to be healthy; the crural artery of the affected side at the groin felt hard, and was no doubt ossified.

The patient had no idea whence his disease arose; he had received no bruise; and his health had been previously very good.

Dupuytren being satisfied that this was a case of the senile gangrene induced by an obstruction, partial or total, of the arteries, in consequence of an inflammation of their inner coat, and a consequent deposition of coagula in their tubes, ordered a large venæsection; and emollient cataplasms at the same time to the toe. The patient experienced immediate relief, and slept comfortably that night.

A mild antiphlogistic treatment was continued, and in the course of a few weeks the eschar was thrown off, and the ulcer, although deep, shewed a beginning granulation. At the date of the report, every thing was going on well.

*Observations.* Medical men have hitherto pinned their faith too undoubtingly to the doctrines of Mr. Pott on the subject of the gangrena senilis;—indeed the very appellation is erroneous. Dupuytren has seen the disease in a girl of ten years of age, and in this case it extended up one half of the leg.

This eminent surgeon, after much patient observation, is now convinced that the gangrene is the result of a previous arteritis of the principal vessels of the affected part. Although it most generally commences at the very extremities of the fingers and toes, he has seen it appear, first of all, in some other part either of the foot or leg, as about the malleoli; and authors have described similar appearances on the trunk, on the nose, ears, &c. The upper extremities are not unfrequently affected, and Dr. Paillard mentions a case, in which the hand and lower half of the forearm had become shrivelled and dry, as we see in a mummy. The early symptoms are a feeling of stiffness, cold, weight, and heaviness in the part; then of tickling and pain, which is sometimes very lancinating and severe; the part becomes swelled and puffy, and of a purplish hue; at other times it is pale and shrivelled. One or more phlyctæna form, and when these break, the subjacent parts are found to be gangrenous, and, as it were, mummified. If the strength of the patient holds up, the disease gradually mounts higher and higher up the limb, and each step of advance is denoted by a repetition of the symptoms just now mentioned. As, is well known, by far the greater number of cases occur in old subjects; and hence probably the cause of ascribing the disease to ossification of the arteries; but this is now known to be, as Hodgson justly observes, only a coincidence; in truth, mere ossification of the blood-vessels does not very materially affect the circulation; and every anatomist is aware how frequent such a change of the arterial tissue is in old people, without being accompanied by any traces of gangrene of the parts. The fact, too, of the disease occurring sometimes, although rarely, in young persons, corroborates this opinion.

The real and essential cause is an obstruction to the circulation ; and this obstruction is produced by the vessels being clogged up with coagulated blood in consequence of the previous inflammation of their serous coat. Morbid anatomy has shewn the truth of these positions most satisfactorily. On examining the vessels, we find that the arteries are filled with coagula, which adhere closely to the tubes by means of coagulable lymph, which has been poured out, as happens so frequently with all serous membranes. These phenomena may, it is true, take place in ossified as well as in healthy and normal vessels, and not in one set only to the exclusion of the other. Cruveilhier has shewn that we may induce arteritis by injecting acrid substances into the vessels, and that mortification of the parts is a frequent result, in consequence of the circulation being interrupted ;—by keeping this in mind, we can readily explain how the dead parts should become dry, withered, and mummified, and not putrid and boggy, as after an excessive inflammation.—How satisfactorily also can we now understand the occasional exciting causes of this malady ; viz. excess in wine and spirits, rich living, certain poisons, especially the ergot of some grains ; the blood becomes infected, and stimulates the arteries to inflammation. M. Roche has written an admirable paper supporting these views in the Dict. de Med. et Chirurg Pratiq. t. vii. art. Ergotisme.

Andral adds his influential testimony to the same effect. Consult his Precis d'Anatomie Pathology, t. ii. p. 373. Delpech, in a paper published in the Revue Medicale for July, 1831, admits that this sort of gangrene is the consequence of an obliterating inflammation of the capillary vessels.

*Treatment.* Every one is acquainted with Pott's curative means, bark and opium : the former is not only inefficacious, but is absolutely hurtful in most cases, and our experience confirms this condemnation.

An old woman, upwards of 60 years of age, was taken into the Hôtel Dieu, many years ago, and treated on Pott's plan ;—the disease gradually extended itself. Dupuytren then ordered bleeding and other antiphlogistic means ; and speedily the mischief was arrested. Since that time a great number of cases have been similarly treated with most gratifying success. Opium is an admirable adjuvant, but it cannot be trusted to alone.—*Trans. Medicales.*

---

### XII. Pommade, to prevent the Hair from falling off.

Take of ox-marrow...... 6 drachms.
Almond oil...... 2
Powder of red bark 1

Add the powder gradually to the oil, blending them well together ; then having melted the marrow over a gentle fire, mix and stir it with the other ingredients in a mortar, until it all becomes cold. Flavour it with any grateful aromatic.—*Bullet. de Therapeut.*

---

### XIII. Extracts from German Literature.

In our last number we noticed some excerpta from modern German works, published in our esteemed contemporary of Dublin by Dr. Robert Graves, and we took occasion to observe that this gentleman was doing a service to the profession in this country by making them acquainted with what would otherwise be to them a sealed book. The paper we are about to notice was published before that which we alluded to in our last number. It is contained in our contemporary for May of the present year.

1. *A wonderful Heart.*

This wonderful heart (cor illud mirabile) occurred in a woman who had been subject to occasional attacks of rheumatic head-ache and arthritic pains, but had otherwise enjoyed good health until some years before her death, when she became subject to a perpetual feeling of anxiety, often accompanied by palpitations of the heart and sudden fits of debility, preceded by transitory glows of heat.

On dissection, a large quantity of water was found in the chest.

"The heart, before the sac of the pericardium was opened, appeared natural in its size and position, but on cutting the pericardial sac, both the ventricles and auricles seemed somewhat smaller than natural. The external or fibrous layer of the pericardium was normal in its texture, whilst the serous layer by which it is lined internally, appeared much thicker than usual, and had altogether lost its natural transparency. That portion of the serous layer, which is reflected over, and embraces the heart itself, was neither thickened, nor otherwise altered in structure, and, as usual, adhered intimately to the subjacent substance of the ventricles, which were found degenerated into a fatty mass. The external surface of this reflected serous layer, was covered with *striæ* and *flocculi* of coagulable lymph, superimposed in a laminated manner, but easily removable, so as to expose the serous layer covering the substance of the ventricles just spoken of.

Between these two serous layers, viz. that lining the fibrous sac of the pericardium, and that reflected over the substance of the heart, was found a stratum of perfectly muscular substance (massa omnino muscularis). The extent of this muscular stratum answered exactly to that of the reflected serous layer of the pericardium, and, consequently, it covered the whole body of the heart, ventricles, as well as auricles, extending from the great vessels issuing from the base of the heart to its very apex. In estimating the relative actions of the muscular fibres composing this layer, it is necessary to recollect that on the different sides of the heart they ran in different directions, imitating in the most perfect manner the natural spiral course of the muscular fibres of the heart, and forming towards its apex a junction by means of a *vortex* of muscular fibres. When examined by a powerful microscope, these fibres were found to be composed of separate fasciculi, each again containing distinct minute bundles or parcels of still smaller fibres, just as is observed in true muscle. The muscular fibres were strong and powerful on the posterior face of the left ventricle, but still more so where they embraced the origin of the inferior vena cava, like a sphincter muscle."

We cannot but suspect that both Dr. Leowolf, of Heidelburg, the narrator of the case, and his translator, have attached more importance to it than it merits. It appears to us to be an instance of chronic pericarditis, and the reputed muscle to be nothing more than lymph, organized perhaps, but presenting appearances familiar to those accustomed to post-mortem examinations. A great deal of speculation on the extraordinary muscular apparatus is appended; but taking the view which we do of the matter, we think it somewhat unprofitable.

2. *Excision of the Articulating Ends of Bones.*

An enumeration of the operations of this description is presented by Dr. Leowolf. It can only be considered an approximation to the truth, indeed, it would seem in several particulars extremely incorrect. We refer the curious to our contemporary.

3. *Hypertrophy of the Mammæ.*

This affection has attracted little notice. Yet it is not very rare, and we have heard of a case in which it was indirectly a cause of death. This case occurred at St. George's Hospital, and was under the care of Mr. Brodie. The patient, a young woman, came up from the country on account of an enormous enlargement of the breasts, without any decided change of structure. The integument was accidentally abraded by a pin, erysipelas supervened, and it proved very rapidly fatal. As this is a disease (it may justly be considered such) of which little is generally known, we are induced to notice the instances collected by the industry of the German author.

*Case.* A young woman, of a pale countenance, slender form, and phlegmatic temperament, had enjoyed an uninterrupted state of good health until her 25th year, when she became pregnant. It is remarked that her breasts were naturally large and soft. No unusual occurrence followed delivery, except that the child could not be brought to take the breast, and consequently the mammæ became distended with milk, and far exceeded their natural size. It is not stated whether they had regained their usual dimensions before she again became pregnant, about two years afterwards. Be this as it may, both had attained to such a magnitude before the sixth month of pregnancy had elapsed, that she sought medical aid, and informed Dr. Cerutti that about four months previously she had received a blow on the *right* mamma, shortly after which the *left* breast became evidently larger; but its enlargement was unattended by the least heat, pain, or any other symptom of local inflammation. In the course of a few weeks the right breast began likewise to increase in size, but not so rapidly as the left. When first examined, they were so greatly enlarged and heavy, that their weight alone proved a serious incumbrance to the patient; they were both equally hard, and the strongest pressure on them did not produce the least pain. The skin covering both was perfectly natural, and she complained of no uneasiness except occasional stitches darting through the left mamma. The enlargement of the breasts continued to increase until the end of the eighth month, when she was delivered of a dead child on the 15th of March, after which their size remained stationary, and the stitches in the left breast ceased altogether. After some time she commenced an alterative course of mercurials and antimonials, which seemed to improve her general health and made some impression on the breasts, for the right was evidently diminished in size. Both, however, were still hard, but in some spots the hardness was so far diminished as to yield somewhat to the finger when pressed. Under the same treatment these soft spots seemed to increase in size and number, and at last imparted an evident sense of fluctuation to the finger; at the same time her lower extremities, and afterwards the integuments of the abdomen, became œdematous, and in the course of a few days even her face and hands were somewhat swollen, particularly in the morning. The appearance of the œdema was accompanied by febrile symptoms, which, together with the anasarca, speedily yielded to antiphlogistic treatment. The left mamma had now become quite soft in every part, and in fact felt like a bladder full of water; its weight and the disagreeable fluctuation of the contained fluid rendered it extremely inconvenient to the patient, and accordingly it was resolved to let out the fluid, which was effected by means of scarifications made on the 14th of April, and repeated eight days in succession. This afforded exit to several pints of water, and caused so great a decrease in the size of the left mamma, that it was no longer much larger than the right. The flow of water through the wounds continued for several weeks, until, indeed, the appearance of the left breast was so entirely altered, that it now resembled a flaccid nearly empty bag, containing the mammary gland somewhat increased in size, and of a stone-like hardness. The alteration in the left breast was less perceptible, nor was it evident that it ever had contained any water; like the other, however, it too had become more flaccid. In neither did she feel the slightest pain even on pressure. The use of iodine ointment, and other remedies, had before the end of July effected a still further reduction in the size of her breasts, which, although still much larger than those of other women, and still exhibiting a remarkable hardness of the mammary glands, yet formed no serious impediment in the performance of her usual occupations. So matters continued for thirteen months, when she became a third time pregnant, and in the course of a few weeks the breasts again began to increase in size, and that with such rapidity, that in the beginning of the following April, the left breast presented the following measurements: circumference at basis, forty inches; from nipple to upper border of tumour, twenty-seven inches; to lower, sixteen inches; the right breast measured an inch less in each direction. These enormous tumours hung pendulous over the abdomen, and entirely prevented her pregnant condition being remarked by the eye, although she was within six weeks of her confinement. In some parts the skin, hitherto natural, seemed distended, ready to burst, and painful. The success of the scarifications on a former occasion, induced her medical attendants to try them again, but it was now found that very little fluid came from the wound, which immediately became gaping, and exhibited a protrusion of the parenchymatous substance of the breast, firm and fat-like, which protruding portion rapidly increased in size, until it resembled a steatomatous tumour as large as a goose egg. The size of the breasts continued to augment daily, and before the period of accouchement, which happened on the 10th May, 1828, they certainly must have together weighed twenty-four pounds. Their heat was above the natural standard, and here and there their surface was traversed by turgid and swollen veins. They were every where elastic, and in no part uneven or rugged from the occurrence of knotty tumours or hard spots. The integuments were more distended towards their inferior and most pendent portion, on account of the gravitation of the fluid to that part.

In consequence of this the inferior parts yielded much more to the finger when pressed, than the superior, and imparted more of the feeling of softness, but nevertheless they did not pit even on strong pressure. The breasts were narrower at their basis than in other parts, and consequently had a pyriform shape; by pressing strongly against each other they had occasioned mutual excoriation and ulceration on their internal surfaces. At the end of her pregnancy another tumour appeared in the right axilla, about the size of the fist. This was at first painful, soon softened, suppurated, and broke; notwithstanding these various sources of irritation, her general health appeared unaffected, and there were no pectoral symptoms of pains whatsoever. A few days after delivery, the breasts began to diminish in size, and in the course of a week the diminution had so far advanced, that the skin covering the tumour, instead of being distended, was wrinkled and loose; for some weeks before and after the birth of her child, the patient was prevented from sitting up in bed, by the pain in her breasts which the change from the horizontal posture occasioned; when it was absolutely necessary for her to sit up or stand, she could only effect it by aid of persons employed to support her breasts with their hands; and when she remained for any length of time sitting, she was obliged to draw her knees upwards, so as to give support to the breasts which hung over and covered the whole abdomen. In a few days after her accouchement she obtained much relief from the bursting of the abscess in her armpit, which discharged a very large quantity of a white, ropy, milk-like, fluid. On the 30th of June, she was able to follow her usual occupations, and although the breasts were still uniformly hard, and so large as to hang far downwards over the abdomen, yet they were amazingly diminished in size, and the integuments covering them hung loosely and in folds. She could then lie comfortably on either side, and suffered no pain; although still emaciated, she was in other respects healthy. On the 7th of September, she applied for assistance on account of the ulceration between the breasts which had never healed, and on account of the non-appearance of the menses since her confinement in May; she appeared pale and cachectic. Our author determined to try the effects of animal charcoal,*

* Animal charcoal has been strongly recommended by Dr. F. A. Weisse, in indurated tumours, scirrhus, &c. &c.

which was administered in doses of half a grain, gradually increased to a grain and a half three times a day; in the course of a month the size of the breast had considerably diminished, and the ulcerated parts had assumed a much healthier appearance, and were healing. Various circumstances, however, prevented her from attending the dispensary, and consequently all remedies were laid aside. She was again examined on the 17th May, 1830, when the left breast, which was still somewhat the larger of the two, was found to measure 21 inches in circumference at the basis, and 9 inches from the basis to the nipple. The substance or parenchyma of the breasts is soft, and the integuments are quite flaccid, loose, and pendant, so as to afford proofs of the former enormous size of the parts they covered.

The foregoing case was insusceptible of abbreviation, it having been already compressed into as portable a shape as possible.

Galen appears to mention excessive enlargement of the breasts. Scaliger briefly describes one; so does Bartholinus. Palmuthius relates a case. A woman had breasts rather larger than usual before her marriage, but their size increased greatly during her first pregnancy, and each succeeding one, until at last they hung down as far as her knees.

A case is related in Welser Augsburgh Chronicles. A servant maid was so incumbered by hypertrophy of the mammæ, that she could scarcely either stand or walk; in every other respect her health was good. The left breast was successfully amputated by a barber, and was found to weigh twelve pounds; the young woman was so relieved by its removal, that she was able to support the burthen of the right breast without any great inconvenience. A lady near Koningsburg had a similar affection; her breasts were so enormous, that one of them alone weighed nearly thirty pounds, and the patient was obliged to have recourse to suspensory bandages tied round the neck to enable her to support them. In this lady the removal of a suppression of the menses under which she had long laboured, was effected by judicious treatment, and an immediate diminution of the size of the mammæ was the consequence of the restoration of a healthy state of the menstrual discharge. A lady of rank who had previously enjoyed a most uninterrupted state of good health, produced a suppression of the menses by incautious exposure to cold during the menstrual period; immediately her breasts became painful, and began to swell, and had so increased in size during the following night, that she could neither get out of bed or move herself. She was twice bled in the foot, and the menses were restored thereby, and the affection of the breasts entirely removed.

Dornsten relates a case. A girl, æt. 20, perfectly healthy, awoke one morning in a fright, and was astonished at seeing both her breasts so enormously enlarged, that their size and weight prevented her from changing her position in bed. The lactiferous ducts were hard and distended, but there was no pain or soreness in the swollen parts. The left mamma measured 31 inches, the right 38 inches in circumference. The attack commenced in July, and in October the young woman died. After death, the left breast, which had continued to grow since July, was cut off; it weighed 64 pounds. It was accurately examined, but presented nothing unnatural in structure, and appeared to be simply hypertrophied. The left breast, which was not removed, must have weighed about 40 pounds. The entire weight of the two breasts was seven stone and a half.

Sauvages mentions a nearly similar case, which occurred at Toulouse. Hey relates the following. A girl, æt. 14, slender, but healthy, was always remarkable for the size of her breasts. The catamenia, which appeared when she was thirteen years old, were suppressed by exposure to cold, and were never afterwards restored; her breasts immediately after the suppression began to grow, and increased in size from day to day with such rapidity, that when seen by Hey their weight was insupportable; amputation of the left breast was performed, and in a short time afterwards, the catamenia re-appeared, and became regular. The remaining breast now began to diminish in size, and in six months was not more than half as large as formerly.

### XIV. Adhesion of the Placenta.

When the usual means for promoting the expulsion of the placenta have failed, some practitioners have recommended the injection of cold water through the umbilical vein.

This may act in two ways; either by stimulating the uterus, and thus exciting it to regular contractions, or by distending the cellular tissue of the placenta, and with it the constricted portion (we are talking of cases of the hour-glass contraction) of the womb. Before resorting to this expedient, we should try the simpler one, of merely immersing the extremity of the cord into cold water; the impression of the cold is thus transmitted to the walls of the uterus, and may induce them to sudden and healthy contractions.—*Archives Générales.*

### XV. Gonorrhœa communicated by swallowing the Discharge.

Dr. Tazentre, of Paris, lately reported the following case to the Faculty of Medicine. Our readers must judge for themselves of its credibility.—*Ed.*

An old sailor, not more chaste after than before marriage, had contracted a gonorrhœa; and as the rogue suspected his wife of infidelity, but had no direct proof of it, he could think of no other way of finding out the truth but by giving her the clap, and then charging her with having infected him. However, the lady was no fool; for observing that her dear moiety was not looking well, and probably having some other good reasons, she declined her husband's affectionate embraces!

Jack was now rather nonplussed; but remembering that, in the colonies, he had learned the art of clapping and poxing, by mixing the discharges with the food of the victims, he now resolved to make the experiment upon his wife. He had repeated it for eight or ten days, when, being caught in the act of stirring something about in a bason of milk, intended for her breakfast, and threatened that it should be taken to a chemist to be analysed, the scoundrel disclosed his infamous practices.

The woman returned to her father's house, and Dr. T. was consulted. At this time, however, no trace of any running existed; but, in four days afterwards, an "intense blenorrhagie" made its appearance.

The author is aware that many who read the above case, will be inclined to attribute to some other cause than to the vile tricks of the husband the vaginal discharge of this woman, who, it is confessed, was visited by another man. He, indeed, was quite sound, Dr. T. informs us.

Dr. T. remarks, in the way of illustration, that many cats, in the "Hôpital des Veneriens," have shewn the symptoms of syphilis, as ulcerations, exostoses, &c. after having eaten the charpie which had been applied to chancres and open buboes.—*Archives Gener.*

### XVI. On the different Sorts of Goitre.

Dr. Sacchi, the chief surgeon of the hospital at Treviglio, has written a very able memoir on this subject, in the December Number of the Annali Universali, from which we shall make a few extracts.

The first form, or species, is that wherein the gland is simply enlarged in volume, but not changed in structure; it has been called by some the fleshy goitre; Dr. S. prefers the term of hypertrophy of the thyroid gland. It is common in young girls and in women—has a regular, even surface, an uniform resistance, and seldom presents any distinct divisions, or lobes.

It may be often cured by medical treatment. If not dispersed, the gland becomes in time variously altered;—

these alterations may be reduced to two leading forms; in the one, the goitre assumes a scrofulous character; in the other, an encysted, or, as it has been called, a lymphatic character. The scrofulous goitre attains often an immense size, but does not give rise to corresponding inconvenience or danger —it is generally lobulated. Now, in course of time, one or more of these lobes may become soft, and give to the finger the feeling of fluctuation; this constitutes the soft, hydatidic, serous, or lymphatic goitre of authors; the structure has become vesicular, and the contained fluid is sometimes watery—at other times mucous or albuminous, like the white of an egg. In a few cases, it is more like milk or pus, or different cells may contain different sorts of fluid. It must, however, be well remembered that some goitres, which have a most distinct fluctuation, yet contain no fluid; the structure of the gland has degenerated into a mass like that of the placenta, or of a wet sponge.

This variety of goitre is remarkably smooth, uniform, and elastic to the touch. Some goitres undergo a partial ramollissement; for it is quite a mistake to suppose that they always become harder and harder, the longer they exist. From what has been stated, it may justly be concluded that hypertrophy, scrofulous change, and lymphatic degeneration, should be considered as three progressive stages of the same disease; and it is not unfrequent to find different parts of the gland simultaneously affected with these three diseased conditions.

It has been a subject of dispute, whether the thyroid gland is ever primarily affected with true scirrhus. Scarpa said *not;* and maintained that the disease was always consecutive to cancer or scirrhus of the tongue, œsophagus, parotid, or submaxillary gland, &c.

Dr. Sacchi has, however, narrated a case in confirmation of the opposite opinion: and the dissection of the tumour must preclude any attempt to gainsay its nature. An example of genuine fungus hæmatodes is also detailed.

One of the most curious alterations of the thyroid gland is that which has been called the aneurysmatic goitre; it is formed by an abnormal or excessive development of the thyroid arteries, and of their branches; the former sometimes acquire the size of one of the carotids. On examining the tumour during life, it is found to have strong pulsations at every point; but the pulsations do not resemble those of an aneurism—they convey to the hand rather a sensation of the blood flowing along very rapidly into numerous vessels, and are accompanied with a sound like an obscure buzzing, or tremulous murmur of the whole surface; but this is more distinct and strong over the site of the thyroid trunks. In two cases, given by our author, the tumours had existed for a number of years, and both had been originally brought on by the efforts of the women during their accouchements.

In addition to the preceding forms of goitre, we may state, that the thyroid is occasionally the seat of tuberculous and melanotic depositions, and of hydatidic, atheromatous, cartilaginous, bony, and even of chalky formations. Now all these, as well as the preceding tumours, are included in the general appellation of goitre. Dr. S. adheres to the old opinion that this disease is very frequently, perhaps most commonly, induced by the prolonged use of unwholesome calcareous waters. In proof of this, he alludes to the sanative results of changing the residence of the patients. This, he says, is by far the most important of all remedial means. Iodine is useful chiefly in the hypertrophic and scrofulous forms; less so in the lymphatic; and is quite inefficacious against the small, isolated, and hard goitres. The best mode of using it is by friction, with an ointment of hydriodate of potass, to be continued for one, or for several months.—*Annali Universali.*

---

### XVII. Anencephalous Monstrosity.

A fœtus born between the 5th and 6th months of pregnancy, presented the following irregularities of development. The arch or vault of the cranium was flattened to a plane surface, on a level with the orbits, and of the occipital hole; from the vicinity of which there projected an immense globular tumour, filled with a liquid cerebral pulp, and with blood; it hung down as low as the lumbar vertebræ. The skin over the nucha and on the back was split, or divided from the occiput to the loins; the trapezii muscles were separated from each other by the prominence of the spinal column. On examining the cranium, it was found that its vault was so depressed, that the point of the finger could only with difficulty be introduced between it and the base. The ossa frontis, parietalia, temporalia, and occipitis, were ossified as much as in a full grown fœtus; they were very small, and joined together by a firm cartilaginous structure, so that there were no fontanelles. The os occipitis was reduced to a large ring of bone; through which the tumour had escaped. The basis cranii did not present any of the normal cavities, or fossæ, for the lodgment of the lobes of the cerebrum and cerebellum. The only vestige of brain was a small organised cerebral pulpy mass, situated in the centre of the base; from this sprung the olfactory and optic nerves; the other cerebral nerves appeared to issue from the base of the tumour; they were soft, easily torn, and of a greyish-red colour. On opening the thorax and abdomen, a complete "bouleversement" of all the organs was discovered, the small intestines were found in the left side of the chest; and the liver and heart in the right side. The left lung was placed crossways from left to right; the right one was shrivelled to very small dimensions, and was pushed up to the top of the thoracic cavity. The heart was much enlarged; the left side turned forwards, and the right side to the vertebræ; the left auricle was wanting, the right one was greatly increased in size; the left ventricle was very small, and without mitral or sigmoid valves; it communicated with the right auricle. The right ventricle much enlarged, was unprovided with any valves, either at the mouth of the auricle, or of the pulmonary artery. The aorta gave off, at its arch, first the right carotid, from which sprung the right subclavian; then the left carotid, the canalis arteriosus, and terminated in the left subclavian; the pulmonary artery having mounted up as high as the first rib, curved round, and ran downwards along the left side of the spine. Immediately after issuing from the ventricle, it gave off large branches to the lungs; it then joined with the canalis arteriosus; and afterwards having supplied in its course the mesenteric, hepatic, splenic, renal, and crural branches, it terminated in the umbilical arteries. The right auricle received all the blood of the body; a small portion of this was discharged into the left ventricle; and the rest, by far the greatest part, into the right ventricle; from which it was sent to the lungs, and into the arteries of the inferior half of the body; the left ventricle received the contents of the pulmonary veins, and propelled these, blended with the blood which had been sent from the right auricle, into the aorta, and thence into the carotids and subclavians.

The particulars regarding the abdominal viscera we must omit. M. Sanson, in his report upon the preceding case, made some interesting observations. He alluded to the deformities of the cranial bones, to the partial destruction of the brain, to the separation of its constituent parts, or ganglions of formation, coinciding with the integrity of the peripheral nerves, and thus furnishing an argument in favour of the respective independance of all these parts, and shewed that these irregularities of structure belong to that kind of monstrosity, which M. St. Hilaire has called "notencephalic."

It is an axiom which, of late years, has been much insisted upon by philosophical and comparative anatomists, that the organism, even in its anomalies, obeys certain immutable laws; and it is said that the imperfections of organization, in one of the higher animals, always correspond with, and constitute the natural condition of these abnormal parts, in some animals lower in the scale. Now, without alluding to the atrophy of one of the lungs in the above fœtus, (a state of things which associated it to the "ophidiens unipulmonés") nor to the absence of the left auricle and the diminished size of the left ventricle, the organ being thus in some degree brought to the state of a simple, or one-sided heart. We shall content ourselves by saying that the almost complete want of the diaphragm, and the huddling together of the thoracic and abdominal viscera, present very striking analogies to the normal organization of reptiles. The analogy is yet more surprising in the circulatory apparatus. M. St. Ange has lately shewn that in the crocodile, the head and fore extremities alone receive pure unmixed blood ; all the rest of the body is supplied with blood, half arterial, half venous. Now, such an arrangement exists in the present case, and is one of the most curious and instructive characters of its history.—*Transact. Medic.*

---

### XVIII. Clinical Observations, by Dr. Steinmetz of Pyrmont.

### 1. *Croup.*

The inefficacy of the ordinary treatment by leeches and calomel, induced Dr. S. to trust almost entirely to the steady employment of emetics. The usual formula he has used is three grains of emetic tartar dissolved in an ounce of water, to be sweetened with sugar. A dessert spoonful may be given at first, and then every five minutes a tea spoonful, until very free vomiting be induced. He has sometimes added three grains of sulphate of copper, in cases which required immediate relief. The nausea and occasional retching should be kept up till the danger be over.

The success which Dr. S. has derived from this treatment is stated to be very gratifying.

### 2. *Excision of the Clavicle.*

A man, aged 31, had been afflicted with scrofulous tumours and abscesses for many years. One of these had formed over the right clavicle, and when it broke, the bone was discovered to be extensively carious. The patient's health becoming gradually more and more infirm, and the local malady not improving, it was determined to extirpate the diseased clavicle.

This was easily done by a cautious dissection. Although very little blood was lost, the patient was exceedingly exhausted, and required active stimulants to restore him. The wound was quite closed in seven weeks, and the use of the arm was eventually little impaired. In the place of the removed clavicle, a firm cartilaginous deposit had been formed : it was flattened at its sternal, and more rounded at its acromial extremity ; and here a few long nuclei were observed upon dissection, the patient having died of consumption, four or five years after the operation.

### 3. *Morbus Coxarius—Guinea-worm about the Hip-joint.*

A coachman having injured his hip against the shaft of a carriage, continued to suffer at first stiffness, and then severe pain in the joint for a length o1 time. In spite of all the means used, several abscesses formed in the neighbourhood ; the general health decayed, and the patient was afflicted with severe attacks of tetanic convulsions.

One day, the surgeon, when he was pressing out the matter from one of the abscesses, observed a foreign body, like a dirty grey string or filament in the wound : upon touching it, it drew back, so as almost to be concealed. By cautious pulling, the surgeon coiled out two inches and a half of this worm-like body, and secured it round a probe to prevent its retreat. Six hours afterwards he drew out the worm entirely ; it measured three-fourths of an ell, was of a dirty white colour, of the thickness of a small quill, or of an earth-worm, not annulated, but uniform throughout ; the tail ended in a blunt point. The mouth and intestinal canal were quite obvious. It died very soon after being extracted. The patient speedily recovered, and ultimately was restored to perfect health.

4. *Monstrosity—Absence of the External Ears.*

In a child a year and a half old, observed by Dr. S., there was a total want of the external ears, except two or three small cuticular elevations, not provided with cartilage, which perhaps may be regarded as imperfect lobuli, although situated higher on the side of the head than the corresponding parts of a normal ear. The sense of hearing did not however seem to be impaired. Was this effected through the Eustachian tube, or in consequence of the external skin acting the part of the membrana tympani?—*Graefe's & Walther's Journal.*

---

XIX. RHINOPLASTIC OPERATION.

A young man consulted M. Dupuytren, some time ago, respecting an eating ulcer which had already destroyed a considerable portion of the point and septum of the nose. Under mild treatment the sore was healed; but a disgusting deformity remained in consequence of the loss of substance.

M. D. resolved to attempt its restoration; and in this case he cut the flap from the upper lip, which was unusually thick and long. Having accurately marked out the dimensions required, an incision was made through one half of the thickness of the lip, and the flap was then dissected or sliced off, the inner surface or face of the lip being left uninjured. The flap was now "retourné" by twisting its pedicle from right to left, and secured to the raw edges of the nose by hair-lip pins, and the twisted suture. Small plugs of lint introduced beneath served to support it. The wound in the lip was then brought and kept together by the same sort of suture. On the 6th day the pins were removed from the lip, and on the 9th from the nose. The flap had united to the septum and point of the nose.

The new appendage was not however very handsome, for the neck, or pedicle of the flap, where it had been twisted round, formed a disagreeable protuberance, and the patient was anxious to be relieved of this annoyance. The Baron would not consent to do any thing, as he expected that it might become smaller and smaller in course of time. Upwards of a year having elapsed, and the deformity being little changed, it was deemed adviseable to accede to the patient's wishes: the pedicle was divided, and all the irregularities pared carefully away. The cure ultimately was a most satisfactory one —*Journ. Hebdomad.*

---

III.

### Institute of France.

I. ACADEMY OF SCIENCES.

*Séances in May.*

DIVISION OF THE SYMPHYSIS PUBIS.

M. Baudeloque has lately performed this operation with perfect success, both to mother and child.

CHEMISTRY OF THE SERUM OF THE BLOOD.

M. Boudet has of late been continuing his curious researches on this interesting subject. His method consists in evaporating the serum to dryness, pulverising it, and after washing it with hot water, subjecting it to the action of alcohol. He has thus been enabled to detect the presence of cholesterine; and more recently of another product, which he calls selorine. It is white and slightly nacreous, has neither acid nor alkaline properties, but is reddened, as cholesterine is, by strong sulphuric acid; when heated, it assumes the appearance of an inodorous oily matter floating on the surface of the fluid. It is readily soluble in sulphuric æther; but very sparingly in boiling alcohol. Besides the seroline, M.

Boudet has discovered a fatty, or greasy matter, like that of the brain, and also a peculiar soap substance formed probably of the margarate, or oleate of soda, in the dried serum.

### Medical Galvanism.

M. Palabrat has been engaged for some time past in endeavours to discover the most convenient method of introducing remedial substances into any part of the body, by means of a galvanic current.

That this can be effected is easily shewn by employing such chemical agents as exert a visible and easily obvious reaction upon each other; thus if we lay a compress well wetted with the hydriodate of potass upon one arm, and upon the other a solution of starch, a fine violet colour is immediately manifested upon establishing the circuit; if iodine be used, instead of the hydriodate, it is speedily found deposited upon the starch. It may be said that the substance which is thus invisibly transmitted from one part of the body to another, follows the surface of the skin, and is not conveyed directly through the interjacent textures, which are the moist conductors and firm part of the galvanic arc. M. P. is of a different opinion, and rests it on some experiments, in which he not only well dried the skin of the arms, but covered a part of it with a gum-lac varnish; and yet there was not any interruption to the galvanic phenomena. By proper management, the medicinal substance which we wish to be transmitted, may be caused either to remain in the body of the patient, or to be withdrawn from it. If we desire the former it will be necessary to employ acupuncturation at the same time.

M. P. assures us that he has met with very satisfactory success in the treatment of some cases which had resisted all ordinary remedies; he mentions particularly a case of enormous sarcocele, and one of obstinate quartan fever. In the former, iodine was passed through the tumour; in the latter, quinine was introduced into the system.

The memoir was entrusted to MM. Majendie, Becquerel, and Savart, who are to report upon it.

### Treatment of Facial Neuralgia, with Belladonna Poultices.

M. Deleau highly recommends the topical application of the pulp of the root of the belladonna, obtained by boiling, to the part affected with the neuralgic pain. It is well to continue the cataplasm until a certain degree of "strychnomania" is induced.

The author assures us that he has seldom failed, when there was no inflammatory or organic affection of the nerves existing.

We must persevere in the use of the remedy for some time, if the beneficial effects do not speedily appear.

### *Séances in June.*

### Lithotrity.

MM. Double, Boyer, and Larrey, read their report on M. Civiale's second memoir on the treatment of calculous patients at the hospital Necker. The cases narrated amount to 51, of whom 43 were subjected to lithotrity; and of these, 27 were cured, 10 died, and 7 remained unrelieved. Of the other 8 patients, who were cut, 5 died and 3 recovered. There were only two women in the list; and in both, the calculi were quickly removed by lithotrity. M. Civiale states, that the early youth of a patient is rather a contra-indication against the operation of breaking the stone in the bladder.

### Dislocation of the Humerus backwards.

M. Sedillot, surgeon at the Val de Grace, read a report of a case of luxation of the humerus backwards into the fossa infra spinata, which was reduced a year and 15 days after the accident took place. This dislocation is so rare, that its occurrence has been denied by some. Dessault never saw a case; and Boyer mentions only one.

### II. ACADEMY OF MEDICINE.

### *Séances in April.*

#### TRIPLE URETER.

M. Civiale communicated the particulars of a dissection, in which he found a third ureter. It terminated by an open mouth, in the prostatic portion of the urethra.

#### POISONING BY THE FUMES OF ARSENIC.

A man, who was a manufacturer of the blue pigment used in painting china, and his servant were engaged in boiling a mixture of nitric acid, of cobalt, and of arsenic. All of a sudden the mattrass burst with an explosion, and the room was filled with the fumes. The servant leaped out at the window, and thus saved himself; his master was less fortunate—he was knocked down, and found himself incapable of rising; he lay on the floor, till the servant returned by the door to drag him out. After eight days' most severe suffering he died; his body had become enormously swollen. This was the case with the servant also, but in a less degree; in the course of forty-eight hours the abdomen was as large as that of a woman at the full period of pregnancy. He was taken into the Hôtel Dieu, and derived much relief from purgatives and baths. On the third day after his admission, he passed a quantity of fetid gas from the bowels, and experienced immediate comfort. The tympanitis was gone, and he left the hospital well.

### *Séances in May.*

#### APOPLEXY.

M. Bouillaud reported several cases of apoplexy, in all of which the left ventricle of the heart was simultaneously hypertrophied, and the chalky degeneration of some of the cerebral bloodvessels was present.

The frequency of such cases has induced almost all good physicians to regard the coincidence as a causal, and not merely an accidental phenomenon; the view which they take is, that in consequence of the enlarged size and greater energy of the left side of the heart, the blood is propelled with excessive force against the delicate and easy ruptured vessels of the encephalon, at some point of which they yield, and thus occasion the apoplectic attack.

The author alluded to the greater frequency of such attacks in periods of great public distress and agitation; and the experience of most practitioners will, no doubt, confirm the accuracy of his opinions. M. Piorry stated that, judging from the numerous cases of apoplexy which have fallen under his notice, three-fourths of them, at least, have been coincident with cardiac disease.

M. Rochoux was not inclined to adopt the sentiments of the preceding speakers; he thinks that by far the most frequent cause of sanguineous apoplexy, is a ramollissement of some part of the cerebral mass, and that this ramollissement is by no means always, nor, indeed, even in the moiety of cases, accompanied with any morbid state of the coats of the arteries.

M. Bouillaud, in reply, alludes to the frequency of ramollissement without hæmorrhage, and vice versâ.

#### SOMACETICS IN RELATION TO ORTHOPODY.

It may probably be necessary to interpret these two Grecian words—somacetic, we presume, is derived from "*σωμασκεω*," corpus exerceo; and orthopody, from "*ορθπυς*," recto pede progrediens. The meaning, therefore, is, the employment of certain exercises, or gymnastics, in the treatment of curvatures, and other deformities of the spine, limbs, &c. M. Bricheteau in the name of the commission appointed to report upon a very excellent memoir of M. Pravaz, who has for several years devoted his attention to this subject, expressed their high approval of the different means which he suggested for the cure of these distressing maladies. Much contrariety of advice has been given by medical men on the question, whether the patient, in a case, say of spinal curvature, should be kept entirely quiet and motionless, or whether, on the other hand, some corporeal exercises may not be allowed. M. Pravaz thinks that this has arisen, in a great measure, from not distinguishing general from partial gymnastics. Each case may, and very generally does, demand a special treatment, and it is, therefore, impossible to lay down precise rules which are applicable to all; the great and paramount object is, to call into active use, and to strengthen, one set of muscles, and to render inactive, another set, which act in a direction contrary to the preceding. Whenever and howsoever we can effect this object, we may be assured that we are following a rational therapeutics. Supposing that the deviation is slight, and the symmetrical disposition of the organs of movement is but little affected, then it is advantageous that all the agents of locomotion be simultaneously but gently exercised; for in this way the body will be best fortified, and the incipient irregularity be most effectually obviated; but, when the deformity is greater, and there is well-marked lateral curvature of the spine, and, consequently, an inclination of the chest to one side, general exercise alone may aggravate rather than better the case; and the reason of this is very apparent; the muscles of the two sides do not act symmetrically and with equal force; on the convex side they are stretched, and on the concave they are relaxed. Some writers have recommended us to trust chiefly to mechanical contrivances, which relieve the weak part of the spinal column from the superincumbent weight—others, again, have decried these as worse than useless; but here, as upon most other occasions, the safety lies in the mean. The profession is much indebted to the late Mr. Shaw, for his judicious and scientific suggestions, and M. Pravaz handsomely acknowledges his obligations to the English author. By following the same path of enquiry, he has succeeded in proving, most satisfactorily, that certain sorts of active exercise are admirably fitted to benefit many cases of deformity.

The indication to be kept in view, is to relieve the spine of the superincumbent weight, while the muscles of the limbs and body are permitted free movements—leaping, wrestling, fencing, and so forth, are highly objectionable; instead of these, clambering along a floating cable, or drawing oneself along two cables, stretched parallel to each other, by hanging to them, and pushing the body on by the effort of the arms and legs, or mounting a rope-ladder backwards, and especially the amusement of swimming, are highly recommended. But, as these exercises cannot be at all seasons pursued, M. Pravaz has contrived a very ingenious carriage, which is pushed up an inclined plane by the patient himself, who, lying on his face, and grasping two lateral stanchions, drags his body forwards, and thus impels the carriage at the same time; it then runs down the plane by its own weight. The muscles of the arms and shoulders are thus powerfully called into action. Another very useful exercise is that of balançoire, or see-saw, recommended by M. Clerc, in his memoir on gymnastics; or that proposed by the author, wherein the patient, lying on an inclined plane, turns the handle of a heavy wheel. Boyer had previously suggested something like this. With regard to the balançoire, M. P. recommends that the patients should stand erect upon the ends, and steady themselves by laying hold of two cords from the roof; one of these cords, viz. that which corresponds to the depressed shoulder, should be a little shorter than the other, so that the effort of suspension of the body, at each descent of the beam, falls more upon the affected than upon the other side.

M. Pravaz, differing from many preceding authors, tells us that, in his experience, the catamenia are generally irregular and deficient in females who are kept long motionless in bed, or on the sofa.

### Exstrophy of the Bladder.

M. Velpeau read a report upon a case of congenital vesical exstrophy, and discussed the different theories which have been proposed to explain this deformity. He criticised severely the theory which attributes it to an arrest of development, and deemed that it was much more rational to suppose that it was the result of a pathological change during embryonic life. M. Breschet advocated the theory of, arrested development, and ably maintained that nothing has been more satisfactorily proved than the fact, that the abdomen, and also the intestinal tube, are open cavities in the early stage of fœtal existence.

*Séances in June.*

INOCULATION OF SYPHILIS.

M. Ricord, as is very generally known, has been lately engaged in a course of experiments to ascertain the effect of inoculating with the matter of primary diseased secretions, as from chancres, buboes, and gonorrhœa; and also with the matter of secondary ulcers, as those on the tonsils, on the skin, and so forth.

The result of these trials confirms the old opinion, that the one set of discharges is contagious, and the other is not.

It is right to observe that the experiments were performed on the individuals themselves, who furnished the matter for inoculation. When the inoculation did succeed, a papula was first formed; this gradually became pustular, and in the course of a few days a scab or crust occupied the summit of each. Upon this falling off, an ulcer, having all the characters of a true chancre, was discovered.

When the virus of a gonorrhœa gave rise to these phenomena, M. Ricord is of opinion that there were cotemporaneous chancres, and that it was, in reality, the discharge from them, and not the running from the uninjured mucous membrane, that was at fault.

OPEN FORAMEN OVALE IN AN ADULT.

A man was recently admitted into the Hospital Beaujon, and died there. He had complained of great weight in the head; round the lips and beneath the eyes there was a cyanotic tinge; the pulse was strong, hard, and regular; the impulse of the heart was moderately powerful; no unusual bruit could be heard, and the temperature of the body was unaffected.

Upon dissection, the foramen ovale was so open, that the point of a finger might be passed through it; and around this large opening, there were several other small ones.

LEGACY OF M. PORTAL.

This distinguished physician left a legacy of 12,000 francs to the Academy, for the purpose of instituting an annual prize of 600 francs to the author of the best memoir on "un Sujet Médical éclairé, par des Données Physiologiques et Pathologiques." The subject for 1834 is—"Quelle a eté l'Influence de l'Anatomie Pathologique sur les Progrés de la Médécine, depuis Morgagni jusqu'à nos jours."

NEW ARTICLE OF FOOD.

A person of the name of Bourlet applied to the Minister of Public Works and Commerce, for permission to introduce a new article of food, which he called "sultana bahmia." The Academy was consulted, and two members were appointed to report. The food is prepared from the young capsules of one of the malvaceæ family; viz. the hibiscus esculentis, so called from being eaten in different parts of Asia and America. Although vastly inferior to the ordinary "plantes potageres;" there is no reason for not acceding to the applicant's petition.

CASE OF EXTRA-UTERINE PREGNANCY, OR OF MONSTROSITY BY INCLUSION.

MM. Villeneuve and Evrat read a report upon a very curious case of the above deviation.

A young unmarried female experienced all the symptoms of obstruction of the liver, when she was about 25 years of age. The enlargement of the abdomen went on gradually increasing for six years. A distinct fluctuation could be felt, and upon introducing a trocar, fifty pints of a limpid, lemon-coloured liquid flowed out. Two years afterwards she was again tapped—fifty five pints were evacuated, and a flock of hair was found to have escaped with the serum. Before her death she was tapped a third time, and more hair came out. On *dissection*, an immense cyst was found, occupying a large portion of the abdominal cavity; in it there were two small bundles of hair, enveloped in a greasy, fatty substance, of the size of hen's eggs; attached to one of these was a fragment of bone, and two or three fœtal teeth. The anterior part of the womb had been wasted away; and the os uteri was so small, that a probe could with difficulty be passed through. All the rest of the genital apparatus had been destroyed, or confounded with the cyst. The hymen was present, and the patient had denied having ever had sexual intercourse.

The preceding case was sent to the Academy by Dr. Philip, of Sarlat, as one of extra-uterine pregnancy; the reporters view it as one rather of "monstrosity by inclusion," and as analogous to the cases narrated by Baillie, Bry, Ruysch, and Dupuytren.

M. Capuron stated that one diagnostic mark between these two sets of cases is, that a placenta exists in the former, and is awanting in the latter.

### Miscellaneous.

### I. On Pellagra.

Some years ago, the Austrian government submitted certain questions to the professors of the University of Pavia, respecting that endemic disease of Italy, which of late has so much attracted medical attention; and required of them answers to each individually. Drs. Hildenbrande and Chiappa were selected to draw up the report. We must confine ourselves at present to the subject of the fifth query—"What are the best means to eradicate the disease; or, if that be not possible, to check at least its progress?" The following schedule of propositions was drawn up in reply:—

1. To institute medical commissions, or inspectorships, in the different departments of the kingdom of Lombardy.

2. The inspectors to visit, at stated periods, every house in their district, and to report to the comptrollers all cases as they occur. The most proper periods for such visits are the months of April and of September, as the disease generally commences, or at least is much aggravated in Spring, and abates in Autumn.

3. Suitable hospitals, or "maisons de santé," should be appointed to receive the cases of pellagra as they are discovered. This advice is given, not from any dread of infection, but in order that the patients, who are always the poor and wretched, may at once be supplied with plenty of wholesome food, and not exposed to the scorching rage of the Summer sun. It is remarked that the disease seldom affects the lower classes of workmen and artizans in cities and towns, even although their food be scanty and not of the best quality; and this is attributed to their labour being not so fatiguing, and being carried on within doors. Besides, when they are ill, there are the hospitals for them to go to. The poor peasants have none of these conveniences. They toil like beasts of burden, and are worse fed than they are; and, even in sickness, there is no relief provided for them. Thus, when once they become pellagrous, they seldom recover, unless removed to some city hospital.

4. A simply-written and judicious tract, describing the most approved method of counteracting the disease, and of treating its early stages, ought to be freely distributed among the lower orders.

5. Baths should be fitted up at the public expense, and the lower classes induced to bathe regularly when the slightest symptom of pellagra makes its appearance.

6. Marriages among pellagrous persons should be forbidden. Unfortunately for the efficacy of this enactment, the disease, in most cases, does not attack the young, so much as those at and above thirty years of age.

7. It would be well that public or government bakehouses were established, to secure a supply of wholesome and well-fermented bread for the peasants.

8. The cultivation of wheat, barley, and rye ought to be encouraged, in preference to that of maize or Indian corn (grano turco), which, at the present time, is far too exclusively used by the poor in Italy. The bread made of it is indigestible, ill-fermented, and not nearly so nutritious as that from other grains.

9. The cultivation of the vine ought to be encouraged everywhere.

10. The education of the lower orders in moral, religious, and in general knowledge, ought to be forthwith commenced and zealously pursued.

[Let them but once feel a pride in existence, by breathing the atmosphere of liberty, and then we may hope for a better state of things. The degrading despotism of monks and friars is tenfold worse than that of the bayonet.—EDITOR.]

The general condition, in respect of domestic comforts, might, by wholesome legislative enactments, be materially improved. Agriculture and most manufactures are susceptible of many beneficial changes. The squalid and damp huts of the country people might be converted into clean and pleasant dwellings; their clothing might be made better; and their food more nutritious. Dr. Chiappa strongly and unhesitatingly affirms that the disease is much less dependent upon any local insalubrity of position, than has been generally supposed. No doubt this evil may materially aggravate the calamity, but it does not of itself induce it, and is therefore only of secondary influence. The root of the mischief is to be found in the misery of a scanty and unwholesome vegetable diet, and of hard toil in a burning climate. Let but the landlords be benevolent and humane, and we shall see much less pellagra in future. The work of the peasants in the fields, especially in the spring, when the hot weather sets in, should not be excessive, and not during the sultriness of noon. The morning and evening hours ought to be chosen. If we attend minutely to the symptomatology of the disease, we notice that the skin is first of all affected, then the mucous membranes, especially of the mouth, throat, œsophagus, stomach and intestinal tube; and lastly the whole nervous system. —*Annali Universali.*

II. PETECHIAL FEVER IN ITALY. The symptoms and progress of this fever, which was lately prevalent at Milan, correspond very nearly with what we occasionally observe in the low putrid fever of the crowded and unhealthy parts of London. It is unnecessary, therefore, to dwell upon these; and indeed our chief object in this notice, is to point out the mode of treatment, which, in the practice of Dr. Beccaria, proved so successful, that only six out of a hundred died. We need not remind our readers, that climate and other casualties may have a very important influence on the character of a disease, as well as upon the constitution of patients labouring under it; and that hence it is a maxim of sound therapeutics to keep in memory these circumstances, in estimating the value of any remedial measures.

Dr. B. informs us, that in very few cases were sanguineous depletions required; the patient's strength was insufficient, and symptoms of fatal exhaustion would inevitably have ensued. It was only when pneumonia, or enteritic complications were present, that he ever resorted to general blood-letting. Sometimes the operation was repeated, even to the third time, before the inflammatory state was subdued. As a matter of course we must be guided by existing circumstances as to the amount and frequency of such depletion; still, as a general remark, we again say, that it was very seldom necessary at all. The blood when drawn was usually black and thin; the clot being of a loose texture. Repeated leechings of the temples, cold to the shaved head, and camphorated blisters to the feet were most effectual in counteracting the cerebral symptoms. Frequent washing of the whole body with vinegar and water was of the greatest comfort to the patient: brisk purgatives of jalap and calomel, followed by milder ones, as senna, rhubarb, salts, &c. were quite indispensable; at the same time small doses of the tartrate of antimony, of nitre, or spiritus mindereri, taken repeatedly, induced a very shooting diaphoresis. Some physicians trusted chiefly to large doses of calomel, which they believed to exert a specific action; but Dr. B. preferred the milder and safer plan which we have mentioned. When there were any local congestive or inflammatory complications, he derived much benefit from antimonial refrigerating drinks, from castor oil, manna, tamarinds, ipecacuan, digitalis, and kermes mineral. If, on the contrary, there was great depression of the vital powers, with coldness of the limbs, and general nervous languor, camphor mixture, with a few drops of laudanum, and of tincture of assafœtida, was the most useful remedy; in some such cases the quinine was given with excellent effects. Whenever symptoms of putridity appeared, the decoction of bark, with diluted sulphuric acid, was freely given; and the surface sponged repeatedly with tepid water and vinegar. It is unnecessary to add that the chamber of the sick ought to be kept quiet, cool, and airy.—*Annali Universali.*

### III. Tonospasmia, a new Nervous Disease.

Dr. Semmola of Naples has recently published an account of a very singular spasmodic disease, which he observed in the hospital of incurables there. It occurred in a young man, of an apparently healthy constitution. As long as he remained quiet, without speaking, there seemed nothing the matter with him; but no sooner had he uttered any sound, than he was forthwith seized with violent general convulsions;—this dependance of the spasms on the voice induced Dr. S. to designate the disease "*tonospasmia.*"

If the patient persisted to speak or to cry out, the spasms continued; and, if he ceased, they ceased also, leaving him perfectly well. The muscles chiefly affected were the extensors of the neck, trunk, and extremities; and the character of the spasmodic movements was, that the legs and arms, after frequent and irregular involuntary convulsions, were suddenly extended; the legs being at the same time brought in contact with and pressed against each other, and the arms forcibly applied to the side. The author compares them to the movements of a frog when submitted to the galvanic action. The malady was only of three days duration when Dr. S. saw the patient; and his health was so good, that he was able to follow his occupation of a porter, provided always that he remained quite taciturn.

Fear seemed to be an occasional exciting cause of the convulsions. The proximate cause Dr. S. supposed to be a "hypersthenia, or irritation of that part of the mesocephalon, from which the extreme roots of the recurrent nerves are given off, in company with the nerves of motion," producing an irresistible association, or sympathy between the muscular movements on which the voice depends, and the general convulsions of the body. His prognosis was therefore favourable; and the treatment which he adopted was depletory; viz. bleeding from the arm, and the application of leeches to the mastoid processes. The patient was very speedily quite cured.—*Annali Universali.*

### IV. Case of an Encysted Abscess of the Cerebellum communicating outwardly.

The following very curious case, related by Dr. Scalvanti, of Pisa, is an interesting contribution to the pathology of the brain.

A soldier, aged 23, of a plethoric and healthy constitution, was admitted into the Royal Hospital of Santa Chiara, with the following symptoms, which had suddenly come on; active pyrexia, severe headach, stupor, hard, vibrating pulse, &c. The left parotid was swollen and inflamed. Active depletions speedily restored him; and all that he now complained of was a pain deep-seated in the left ear, accompanied with tinnitus. Blisters and other topical means were tried, but to no purpose; he therefore left the hospital; but soon returned; and now, in addition to the otalgia, there was a swelling of the meatus externus, and he was tormented with head-ache. By cupping, antimonial ointment, &c. he was relieved, and enjoyed a respite for several days; but it was only a respite, for again came back all his distresses worse than ever; the head-ache was accompanied with violent pulsations and a feeling of burning heat; the patient was feverish and watchful, and the integuments over the squamous bone were puffy and inflamed; leeches were applied to the inside of the nostril, with considerable benefit; still there was the beating pain in the head, which at stated periods became much exacerbated. For about six days he was tolerably easy, but this deceitful calm was soon followed by another attack of suffering; the swelling of the integuments had now increased, and pressing them with the finger caused pain, and left a pit.

These alternations of suffering and relief, the distressing head-ache, which never altogether left the poor patient, and the immunity of the intellectual faculties, led Dr. S. to predict disease of the cerebellum, according to the opinion announced by Lallemand in his Anatomico-pathological Researches. A doubt existed, whether the cerebellum was primarily diseased, or subsequently to a disease of the internal ear. However this might be, the man became worse; in spite of occasional intervals of a few days ease, each attack was more severe and alarming; he became almost quite deaf and stupid, and the external swelling extended along the parietal and occipital bones. A surgeon who was called in consultation differed in opinion from Dr. S. and recommended an incision upon the mastoid process. He considered that the disease was altogether external, and that no suppuration of the cerebellum could have taken place, because there were no symptoms of compression, and the intellect was little impaired. He was not aware of the results of Lallemand's researches. The incision was made, and the bone laid bare, but no appearance of disease was to be seen; the lips of the wound were however kept apart. The result seemed at first very gratifying; the head-ache and deafness were surprisingly relieved; and the external swelling much reduced. His physiognomy however became more stupid, and his speech betrayed a wavering state of mind. It is to be observed, that during the intervals of ease, his appetite was always vigorous; unfortunately for himself he on one occasion had indulged to excess; he was seized with obstinate vomiting; became paralytic, and died on the 29th of June.

*Dissection.* On cutting down to the bone, the temporal muscle was found to be healthy; the pericranium was somewhat thickened, and a spoonful of pus was found underneath it, between the squamous and zygomatic portions of the os temporis; a hole penetrated right through the bone, just above the meatus auditorius externus, and over the phrenological organ of destructiveness. The membranes of the brain were highly injected;—that portion of the left hemisphere, which occupies the middle and lateral fossa of the basis cranii, was very considerably increased in volume; the cerebral anfractuosities had disappeared, and the cerebral substance was unusually resistant and elastic; the dura mater was perforated opposite to the hole through the bone. Upon opening the lateral ventricles, it was observed that the left one was sensibly diminished in capacity; and right beneath it, a sac, or cavity of the size of a hen's egg was found; the medullary substance had been wasted away, so that the boundaries of the sac were formed by the cortical or grey portion—it terminated outwardly in a funnel-shaped prolongation, which communicated by the previously-mentioned apertures through the dura mater and the bone, with the abscess under the pericranium. The walls of the sac had a fibrous appearance, and altogether resembled an inflamed mucous membrane. The rest of the encephalon was normal. —*Annali Universali.*

### V. ATROPHY OF THE CONVOLUTIONS OF THE BRAIN IN THE FŒTUS.

[M. Cruveilhier's 17th Fasciculus.]

Although M. Cruveilhier's work has the disadvantage of being extremely diffuse, and unconnected in the mode of its arrangement, there can be no question that it forms a valuable addition to modern medical literature, and that many affections are described in it, which were previously altogether unknown, or very little understood. The notice on atrophy of the convolutions of the fœtal brain will probably admit of being placed in this category.

M. Cruveilhier observes that lesions of the convolutions of the cerebrum and cerebellum are much more frequent than is supposed. He is extremely careful in raising the membranes, and in washing the surface of the convolutions.

Atrophy of the convolutions may be general or partial, congenital or subsequent to birth.

When it takes place prior to the complete development of ossification, and the deficiency of brain is not made up by a liquid, the bones sink in proportionately to the atrophy. If, on the other hand, ossification of the bones is completed, or if, the ossification being imperfect, serum replaces the deficiency of cerebrum, the cranium may maintain its natural volume or even exceed it, as in certain cases of congenital hydrocephalus where scarcely any cerebral matter exists. In some cases the cranial bones, instead of sinking inwards, exhibit an extreme thickness.

Atrophy of the convolutions presents itself under several forms.

1mo. It often consists in a simple diminution of volume.

2do. In other cases it is a sort of shrivelling of the convolutions, which present an unequal and granular surface. Sometimes this shrivelling is accompanied with different shades of colour, evidently dependent on a former effusion of blood. In either case, the serum fills the void between the bones of the cranium and surface of the brain. This serum occupies the subarachnoid cellular tissue, and when the atrophy is very circumscribed it raises the arachnoid, like a serous cyst.

These two kinds of atrophy are often observed in old men whose faculties are impaired; and in old women known at the Saltpetrière by the name of *gâteuses,* because they pass under them both their urine and their stools, are bed-ridden, and usually die of bedsores.

The granular shrivelling of the convolutions very often co-exists with apoplectic atrophy of the same convolutions, and occupies the vicinity of the cellular cicatrices. In this case the surface of the convolutions presents most commonly a yellowish discoloration, attesting the prior existence of a sanguineous collection in the neighbourhood. This shrivelling co-exists also with yellowish points, as small as grains of sand, and with small linear cicatrices cutting the convolutions longitudinally or transversely. These points are so small, the lines so narrow, that they would escape even an attentive examination.

3tio. There is a third order of atrophy of the convolutions which consists in their transformation into a cellular tissue of a brownish colour. This however is the result of an apoplectic attack, and M. Cruveilhier reserves its more complete description until he arrives at the alterations occurring in the sites of apoplectic extravasation.

4to. There is an atrophy of the convolutions with induration of the cellular tissue, which sometimes becomes as firm as cartilage. This induration is the result of inflammation.

5to. Another form of atrophy results from the loss of substance undergone by the convolutions after the red softening. One or other face of the convolutions, often both, are deprived of the grey substance to a greater or less extent. The limits of the loss of substance are marked by an irregular line. The surface of the convolution so denuded is covered by a cellular membrane, of a yellowish colour, and more or less vascular.

6to. A sixth kind of atrophy consists in the transformation of a portion, or of the whole of one hemisphere, or of two hemispheres, into a membrane of extreme tenuity. Often there remains of the brain, only a nucleus represented by the optic thalamus, and the corpus striatum more or less altered. This lesion is most commonly congenital, but M. Cruveilhier is in possession of some cases which appear to shew that a similar atrophy extended to the whole of one hemisphere has been post-natal.

7to. In a seventh kind of atrophy each convolution is converted into a serous cyst with transparent walls, giving at first the idea of an hydatid cyst.

M. Cruveilhier appends a case, but we do not think it necessary to subjoin it.

### VI. On some Diseases of the Muscles.

[M. Cruveilhier, Fasciculus 17.]

M. C. observes that the muscles display all the alterations of tissue which occur in other organs. He enumerates the following:—1mo. Atrophy, one of the forms of which consists in fatty degeneration. We may mention that we saw a remarkable instance of this in a subject brought into the great Windmill Street School for Dissection. All the muscles were converted into a fatty substance, and so wasted that dissection was abandoned. Of the history of the case we know nothing. 2do. Hypertrophy. 3tio. Inflammation and all its consequences. 4to. All organic growths and alterations—the conversion into cellular, fibrous, cartilaginous, or osseous structure, and the formation of cysts of various kinds. 5to. Tubercular and cancerous degenerations, &c. 6to. They may become the seat of spontaneous sanguineous effusions, which have all the character of apoplectic extravasations.

M. Cruveilhier first describes the latter.

*Apoplexy of Muscles.*

At the head of cases of this sort, M. Cruveilhier places apoplexy of the heart, from which cause he believes that the greater number of ruptures of the organ take their rise.

The muscles of animal life do not escape this lesion. In the latter stages of scurvy, we sometimes find in the thickness of the muscles considerable sanguineous collections. M. C. saw a scorbutic patient, who felt an extreme degree of prostration, and whom he persuaded to make an effort to get out of bed, in the hope that a little exercise might be advantageous. Next morning the posterior part of the leg was found enlarged, tense, hard, and of purplish colour. There had been evidently laceration of the muscle, with extensive sanguineous effusion.

It is not uncommon to find sanguineous extravasations occur in the muscles, independently of scurvy. M. Cruveilhier has observed this five or six times in the great straight muscles of the abdomen, which he thinks particularly liable to the affection. A very remarkable instance of this sort occurred in a female, in the poor-house of Limoges. She was affected with a quartan fever, which had proved extremely obstinate. All at once she was attacked with violent pain in the abdomen, aggravated by the slightest contact. Peritonitis was suspected, and a great number of leeches were applied; but the patient died. On opening her body, the two recti muscles were re-placed by coagula, excepting the thoracic portions; the aponeurotic sheath was distended with the clots, in the middle of which the debris of muscular fibres were discovered. Very lately, M. Cruveilhier observed a similar laceration in an individual who died of delirium tremens. M. Rousset, a distinguished physician of Marseilles, has published the case of a man who died on the twelfth day from the invasion of phlegmonous erysipelas. Twenty-nine sanguineous tumours were found in the substance of the muscles. Some were formed by black and partially-coagulated blood—others by blood resembling lees of wine—others by pus mixed with clots of blood; some were made up of creamy pus, and three small abscesses were found in the substance of the heart.

M. Cruveilhier believes that he has demonstrated that sanguineous collections in the muscles have been produced in many animals by phlebitis, the consequence of irritating injections thrown into their veins. The following is an abstract of some of his experiments. He threw, by means of Anel's syringe, a mixture of ink and water into the femoral vein of a great number of dogs. The injection was made in the direction *from* the heart, the valves having been sufficiently broken down by a probe. In the animals who died in the first few days, all the muscles of the lower extremity were studded with sanguineous collections formed by clots deposited in the middle of the torn muscular fibres. In the animals who survived, M. C. has found, at the end of one or two months, cicatrices in the muscles, resembling those seen after apoplexy in the brain. The muscles which had been merely injected with blood, and not torn, presented only the ochrey colour, indicative of former sanguineous extravasation.

M. Cruveilhier thinks that these experiments throw light on a formidable disease, which he has witnessed several times, and which consists in a very intense inflammatory fever, attended with ecchymoses, and sanguineous collections in the greater number of organs, but more particularly in the muscular system. M. Guéneau de Mussy shewed him a very remarkable case of this description in the wards of the Hôtel Dieu. A young man, who had not been exposed to any cognizable causes of scurvy, presented large ecchymoses, with considerable tumefaction in the face, giving him the appearance of having been severely bruised. A large sanguineous tumour existed in the site of the great pectoral muscle, another in that of the deltoid, and others in the lower limbs. These tumours, which did not permit the slightest motions of the limb without excessive pain, were soft in the centre, and very hard at the circumference, like bloody tumours of the scalp. M. C. communicated his ideas on the subject to M. Guéneau de Mussy, and the patient was bled, and treated on the antiphlogistic method. The blood was rapidly re-absorbed, and the patient speedily cured.

### VII. Case of Diaphragmatic Hernia.

[Cruveilhier, Fas. 17.]

A female patient in the Salpêtrière, æt. 75, rachitic, and subject for several years to severe, but temporary colics, was brought to the Infirmary in the following condition :—Skin cold and blue—pulse absent—constant vomiting —abdomen large, and tender on pressure. The absence of diarrhœa distinguished the case from cholera—of an external tumour from external hernia. In two hours after her admission, the patient died.

*Dissection.* On opening the thorax, its left side was found to be almost entirely filled by a large tumour, evidently a herniary one, the transparency of the sac allowing the convolutions of the viscera to be distinguished through it. The stomach, large intestine, and some folds of small intestine, contained in the sac, were of natural colour ; other portions of small intestine were dark and congested, from strangulation. The source of strangulation was not at the neck of the sac, for the finger passed readily through it. There was no bridle or source of stricture within the sac. The constriction proved to be in the abdomen, where the mesentery was twisted on itself, and so applied to the intestine as to cause its strangulation. The diaphragm presented an opening, corresponding in size to the neck of the sac. The heart was much pushed down to the left side.

As M. Cruveilhier observes, the case is curious, in presenting at once a thoracic hernia and an abdominal stricture. There are two varieties of phrenic hernia—the congenital and the accidental. M. C. considers this an example of the former.

A great number of facts have led M. Cruveilhier to the conclusion, that accidental diaphragmatic hernia is formed in this manner only. A mass of fat is formed between the peritoneum and the diaphragm, behind the xiphoid cartilage ; this, gradually increasing, separates the fibres of the diaphragm, and penetrates into the mediastinum ; the peritoneum, dragged by the tumour, follows it, and slowly the viscera succeed.

### VIII. Amtliche Acusserungen ueber die im Grossherzogthum Sachsen-Weimar gegen die Cholera gerichteten Medicinal-polizeilichen Maassregeln von Dr. *L. F. Froriep.* Octavo, pp. 47. Weimar.

Remarks on the Medico-political State Regulations instituted against the Cholera, in the

GRAND DUCHY OF SAXE-WEIMAR. By Dr. *Froriep*.

We quite agree with the able and active author of these pages, as to the utter inefficacy of regimental cordons and of quarantine prohibitions to stop the march of this new-born pestilence. The invisible monster heeds not Russian bayonets, nor British orders of council; but, chuckling at the contemptible impotence of human precautions, it has made almost every nation of the world pay tribute on its altar. Had the opinions which we expressed, before the cholera reached our shores, been attended to, and had our faithful and well-intentioned advice been followed, instead of the panic-struck and panic-striking manifestoes of a Government board of health, a calm, quiet and intelligent preparation would have been made, and much unnecessary expense, and much fatal alarm, might have been avoided. It is not a little gratifying to us, to find that the sentiments of many of the best physicians on the Continent tally with our own; and, without vanity, we may hope that not a few of them have been converted by the perusal of our pages. A salutary lesson has been taught us by the errors which were so abundantly committed last year, alike by professional and unprofessional people; and we trust that our judgments may not be again blinded, either by wavering fear, by interested selfishness, or by presumptuous quackery. The system of issuing a set of medical prescriptions, and of advising every one to keep a druggist's shop in his house, can never surely be repeated, unless, indeed, the chemists and apothecaries be at the helm of government. We have no hesitation in saying, that manifold and most pernicious evils arose from this foolish admonition. In a country like this, above all, in a city like our metropolis, where doctors are almost as rife as patients, the only precautionary measure required was, urgently to recommend immediate application for professional aid; and, instead of erecting hospitals here and hospitals there, and transporting patients, who were frequently moribund, and not a few of whom died before they reached the doors, had but a moderate sum been set aside to remunerate the private medical attendants on the pauper population at their own houses, however uncomfortable and wretched these may too often be, far more efficient aid would have been afforded, and much needless expense been saved.

For example, in one of the healthiest parishes, no fewer than four district infirmaries were established, and in one, if not in two of these, the number of patients admitted did not exceed four or five.

Our friend Dr. Froriep will thus observe that we are not inclined to go so far in adopting precautionary measures as even he recommends; but it is proper to state that his work was published at the very commencement of last year; and perhaps, therefore, his sentiments may have undergone some change since that date. It gives us much pleasure to express our high esteem for his zealous and indefatigable labours, evinced in the work he has sent us.

IX. SYMPTOME DER ASIATISCHEN CHOLERA ZU BERLIN. Von Dr. *Robert Froriep*. Octavo, pp. 90. Weimer.

We have no intention of entering upon an analysis of this work; not that it is in any way deficient in merit, but solely because the task is quite unnecessary. The cholera was precisely the same disease at Berlin, as it exhibited itself in London; and as almost all our readers, both here and elsewhere, have had abundant opportunities of witnessing the real tableau, it would be a waste of time to present them with a written or a pictorial one. Both are very faithfully given by Dr. Froriep; for, appended to the work are eight copper-plates, some of which are exceedingly well done, and convey a very accurate image of the aspect of a cholera patient, before and after death—of the sunken eye, the icy cold tongue, the rumpled half-sodden skin; also of the evacuations by vomiting and by stool; of the urine at the different periods of the disease; and of the morbid appearances found on dissection in the abdominal and pelvic viscera.

The highly injected state of the vessels of the intestines is very satisfactorily portrayed. One of the figures in the 7th plate represents the deep-congestion of the caput cæcum in a case of cholera asphyctica, in which bloody stools had been voided.

Dr. F. describes three forms of the disease:—1, the mild, or, as he terms it, diarrhœa cholerica; 2, the cholera gastrica; and 3, cholera asphyctica. These two latter species are mere shades or degrees of each other; and the only diagnostic symptom between them, according to our author, consists in the pulse being always sensible to the finger in the former, and often not so in the latter. Surely this is not sufficient to establish a difference of species.

Some of our German brethren appear to have carried their scientific curiosity a little too far, when they cut down upon the brachial and axillary arteries, in order to draw blood, or merely to inspect the appearance of the vessels. On opening the tubes, sometimes no blood at all escaped; they were quite empty; or nothing but a little clotted blood was found; and at other times, a few ounces of a red and much changed fluid flowed out at first in a continuous stream, and afterwards in jerks. Dr. F. does not enter upon the subject of the treatment of the disease, which he has so accurately described. This we regret, as it leaves his memoir, otherwise an exceedingly good one, incomplete—like the play of Hamlet, without the character of the prince.

### X. On the Position of the Embryo and Fœtus in the different Classes of Animals.

M. Dubois, in a late interesting article published in the memoirs of the Academy of Medicine, has very ingeniously shewn that the position of the fœtus, the head being always directed towards the os uteri, is quite independent of any influence of gravitation, and he has endeavoured to prove that it results from a sort of instinct of the little one to escape from its prison in the most easy and least dangerous way. He has examined the question, not only with reference to the human subject, but also to some of the other divisions of the mammalia; and the conclusion is surprisingly steady and uniform, that almost always the head of the fœtus is protruded first.

Not satisfied with these data, Mons. Virey has recently extended his enquiries to the other groupes of the animal scale, and has ably demonstrated that the same law seems to operate upon them. In all, or almost all, the direction and position of the fœtus is the same, the head being born first. The frequency of irregularity in this respect is infinitely greater in the human than in any other species, and in civilized than in savage life. Hence it is that in our researches as to the laws of parturition, woman is perhaps the very worst type, or standard of reference, which can be assumed. The extravagancies of fashion and custom, the pernicious effects of dress, the influence of the passions, and many other causes operate upon her frame, but cannot affect that of the lower tribes. Nevertheless, even with her, the rarity of abnormal labour is very wonderful: in an overwhelming majority of cases, the fœtus in utero is placed with its head to the vagina; and as it was known that the head was the heaviest part, the inference was very natural, that therefore it was dependent. Moreover it was found that if a mature infant was suspended by the umbilical cord, the head preponderated downwards; and this was long considered as an argumentum crucis in favour of the explanation given. Even in very modern books we find it stated that, about the seventh month of pregnancy, the head of the fœtus acquires such an increase of size, that the position becomes quite altered; that hitherto it had been uppermost, but that now it falls down, making a complete 'culbute' with its heels up. The objection to this statement was at once obvious; for how can this explanation, by change of relative gravity, be applied in the case of the lower mammiferous animals? In them, although the body of the mother is nearly horizontal, and the head of the fœtus does not so much preponderate, yet the position of the latter is uniformly the same.

It is perhaps almost unnecessary to expose the fallacy of the experiment to which we have above alluded; viz. that of shewing the influence of the superior weight of the head by suspending a fœtus by the cord. Is not the cord in a multitude of cases coiled round the neck? and is not the placenta sometimes attached to the os uteri, and yet the head is dependent, although the point of suspension by the cord be thus altogether changed? The opinion of M. Dubois is, that the fœtus is endowed with an instinctive and unconscious impulse to assume a particular direction, somewhat in the same manner as the magnetic needle mysteriously points to the poles. We cannot however admit the existence of such a blind, vague, and unintelligible agency;—instinct appears to have little or nothing to do with the phenomenon; and the only rational explanation is to refer it to an ultimate law of the living organism. Farther we cannot go. M. Virey assures us, that if we examine female quadrupeds at different periods of gestation, we shall always find the embryos and fœtuses in the cavity of the horns, as well as in the body of the uterus, so placed, that the head is directed towards the vulva. Thus in a bitch, all the fœtuses descending along the cornua or trumpets of the uterus, have their faces pointed to the vulva; one following the other in a row, like a pack of hounds upon one track. The exceptions to this arrangement are very rare. Let us now examine what is the case with the young of oviparous animals. One might naturally suppose that the spheroidal form of the egg rendered indifferent any particular position of the contained fœtus; but not so. At first the two chalazæ, which retain and fix the vitellus by its two poles, keep also the embryo in a uniform and definite position. The head of the chick is very generally turned towards the big end of the egg, which is here more fragile and more permeable to the air. It is this end which is usually directed to the oviduct, and which is first protruded from the cloaca; and as this holds equally with unimpregnated as with impregnated eggs, the conclusion is very obvious—that the position is by no means dependent upon any influence of the chick, but is rather the result of a simple original anatomical predisposition. M. Dutrochet, in his learned report to the Institute, has these valuable remarks. In birds, the ovum in passing from the ovary into the oviduct, preserves a uniform position; the cicatricula, or embryotic germ, is placed equidistant from the two prolongations of the chalaziferous membrane, which the ovum receives in the oviduct; and always on the lightest, and therefore the most buoyant, and superior side of the vitellus, which is divided into two unequal longitudinal hemispheres, by the insertion of the chalazæ. This uniform and constant position of the ovum at its arrival in the oviduct attests a position equally uniform and constant, while it was yet in the ovary; the one is the consequence of the other.

Now the chick, whose embryotic lineaments exist in the cicatricula, having fixed and determinate connexions with the vitellus, it follows that during its development there will be a tendency to assume and to retain a definite position; and this position will be the same in all eggs. It is towards the big end of the egg that this direction of the chick tends; a direction which is evidently the necessary consequence of the original anatomical position of the yolk, or of the ovum in the ovary. The same reflections are applicable to the oviparous, ophidian and saurian reptiles. M. Virey had lately an opportunity of ascertaining the accuracy of this in a female 'viperia aspis.' Upon opening the oviducts he found eight little vipers, already escaped from the shell; all of them were so placed that the heads were directed towards the vent. So much for birds and reptiles.

In fishes it has been repeatedly ascertained by observation, that the fœtal embryo escapes with its head foremost. And if we examine the direction of the scales on the body of fishes and of reptiles, and of the feathers and hairs on fœtal birds and mammifera, we shall find that they are uniformly directed from the head towards the tail when the animals are born. Had the feet of the young one been first expelled, the feathers and scales might have been brushed forward against their natural direction; and thus the escape from the shell might have been rendered difficult. Let us descend still lower in the scale of animals, and even in insects we shall find that the same law seems to be dominant. Every one knows that the larvæ escape head-foremost from the egg, and that the caterpillar eats through its silky envelope, and the crysalis through its shell. The disposition or arrangement of the covers is sometimes so adapted, that the shells of several chrysalides open by a sort of lid at their anterior extremities. The only exception rests upon the authority of Bonnet; he says that the females of the aphis or plant-louse are born backwards "en reculons;" but that the males escape in the usual direction, that is, with the head first. This statement, although confirmed by the observations of Dutrochet, requires further examination, before we can admit its established accuracy.

Among the vermes, we may take the leech for an example; and in its case we know that the young animal escapes head-foremost from its envelope.

We have thus shewn how uniform and steady that law of parturition is, by which the embryo presents forwards or towards the light, its nervous cephalic pole, or extremity; whether the fœtal envelopes are naturally thinner and more delicate at that part where the head lies, or whether the impulsive effort of growth, or the progressive movements of the fœtus, weaken the tunics of the egg there, we are not prepared to say; certain it is, that they rupture more easily at this point, for the exclusion of the fœtus, than at any other. Even in animals which are fissiparous, or which propagate by buds on the body of the mother (and these buds are to be considered as ova, generated and developed at the surface), the part which corresponds to the head invariably makes its appearance first; and if we pass in succession to the vegetable kingdom, a similar law seems uniformly to prevail. We may, therefore, assume that a universal law of organization, and not a result of gravitation, nor of an instinctive impulse of the fœtus, which arranges its position so that the head escapes first; and we must regard the phenomenon as one of those ultimate laws whose existence we may, and should ever, strive to ascertain, but whose cause or antecedent we know nothing about: such likewise is the law of the ascension of the plumule, and the descent of the radicle of an embryo plant. The fanciful speculation of M. Virey, that as the "nervous or exciting element proceeds from the male in copulation, the ova are so disposed that the anterior and superior region of the body, which is the depository of the nervous apparatus of the embryo, must be first exposed to the impregnating influence of the semen or pollen," need not detain us long to examine. It is much more philosophical to rest satisfied with the fact, than to pen such phantastic nonsense. How do we know that the semen of the male is the "nervous element?"

*Conclusions.* 1. That the embryo, while situated either in the ovary, or oviduct, or uterus, always presents, in its normal condition, the head foremost.

2. That this position is purely organic and primitive, anterior to the vivification of the embryo.

3. That the position of the germ or plumule in a grain-seed is analogous, and is dependent upon a similar principle.

4. That the agency of gravitation, or of any impelling instinct, cannot be admitted; as we have seen that the position of the unimpregnated ovum is the same as that of the impregnated one.

But although we exclude instinct in the present case, let not our readers suppose that we deny the existence of all instinctive impulse to the fœtus; on the contrary, we regard many of its spontaneous acts as evidences of such an agency; and one of the most convincing illustrations is, that of the young chick pecking its shell with its beak, in order to make its escape. The seat of instinctive operations we consider to be the ganglionic apparatus of the trisplanchnic nerves, whereas the intellectual functions reside in, or are somehow mysteriously associated with the cerebral mass.—(Vide Histoire des Mœurs, et de l'instinct des animaux, 1821.)—*Revue Medicale.*

### XI. POLYPOUS CONCRETIONS IN THE HEART.

No subject of pathological anatomy has given rise to wider dissensions than that of cardiac polypi. The older anatomists not only admitted their occasional occurrence, but repeatedly describe the general appearances and structure of these masses in their writings. Morgagni, Lieutaud, and Sanac were the first to maintain the contrary opinion;—they alleged that the masses which are denominated polypi are only concretions of the coagulable part of the blood, which for a short time before, and also after death, gradually separated into its constituent parts; but they stoutly denied that these polypi are ever formed long antecedent to death, so as to entitle them to a place in the nosological catalogue.

Corvisart, Burns, Testa and Laennec, high authorities in medicine, have however favoured the doctrine of the earlier anatomists, and have endeavoured to point out symptoms which may indicate the presence of such growths;—they all agree in referring their original or primitive development to fibrinous concretions, which must pass through several phases of condition before they constitute legitimate polypi; but the character or nature of these phases is very differently explained by each of these pathologists. The most widely diffused doctrine upon the subject is, that they are to be viewed as products of inflammation of the lining membrane of the heart, ('carditis polyposa,' of Kreysig,) and are therefore analogous to pseudo-membranes. Whether we admit this opinion or not, one thing is now certain beyond doubt, that living organized polypi may exist within the heart. One of the most satisfactory cases is, that lately communicated to the Anatomical Society of Paris by M. Choisy.

*Case.* A man, aged 47, had suffered during 17 years before his death from almost constant shortness of breath, from cough and violent palpitations;—these symptoms had increased to great severity for five months previous to his admission into the hospital. Although the cough was incessant, no unusual râle was to be heard at any part of the chest; it was uniformly resonant upon percussion; the cardiac region however sounded dull for a much greater extent than natural; and the first sound of the heart was sometimes suddenly interrupted, by a sort of convulsive jerk, or 'saccade;' it seemed, to use Laennec's description, as if a spring behind the heart was suddenly let go, and jerked the organ forwards against the thoracic parietes.* A dry rubbing bruit "de frottement sec et rugueux" was heard between the two cardiac sounds. The pulse was frequent, irregular, and intermittent; and the beating of the heart was felt feeble, and sometimes scarcely appreciable by the hand. The patient became gradually worse; the bruit de frottement became more intense and also so extended that it was to be heard in the pulmonary artery, in the arch of the aorta, and in the subclavians; general œdema supervened, and death soon afterwards.

*Dissection.* The heart was found hypertrophied to twice its ordinary size. In both auricles there were large sanguineous clots; the one contained in the left auricle enveloped a pyriform body, as large as a partridge's egg; its surface was evidently lined with a continuation of the investing membrane of the auricular cavity, and it was inserted by a fleshy pedicle, four or five lines long, upon the centre of the fossa ovalis—its fundus or base rested upon the auriculo-ventricular orifice, in which it

* The second sound, or that of the dilatation of the heart, was clear and resonant.

seemed to have, as it were, tried to be engaged; it was tuberculated, and unequal or jagged upon the surface; and its consistence was nearly that of a fleshy polypus of the nasal passages. When cut through, it exhibited a texture moderately firm, and of a pale amber colour, except towards the root, where it was more of the colour of the fibres of the heart; the base or fundus of this vegetation was inclosed in a semi-cartilaginous and semi-osseous shell, or covering; and this was cancellated, or divided by partitions into numerous cells, of different dimensions. These cells, the largest of which might scarcely admit a French bean, were filled with a whitish, cerebriform matter, in the centre of which were several firm granulating points.

The left auriculo-ventricular orifice was considerably amplified; its diameter measured at least an inch and a quarter; the mitral valve was wrinkled and tuberculated, and the annulus, or ring of its implantation, was so rough, swollen, and mamillated, where it was opposed to the surface of the polypus, that it resembled not a little the orifice of the anus, when occupied with syphilitic excrescences.

From the preceding detail, M. Choisy is of opinion that he is justified in assimilating this polypous growth with the fleshy productions of the uterus, and with the usual polypi of the nose, antrum, &c.

There are a few analogous cases on record; still it must be confessed that such occurrences are exceedingly rare. The symptomatology of polypus of the heart must necessarily be obscure and unsatisfactory; indeed, we can never expect to discriminate between this and some other morbid affections of this organ. Perhaps, when they are moveable, there may be a variableness and irregularity in the distress or impediment to the circulation, and in the occurrence of the abnormal bruits which arise, such as is not met with in other cases of obstruction.—*Revue Med.*

### XII. On the Bruits of the Arteries and Heart.

M. Bouillaud, in the 144th Number of his Journal, has communicated the results of his extensive clinical practice on the above interesting subject, and we doubt not that our readers will be gratified with a condensed view of this able author's researches.

He states that, if we attentively apply the ear over one of the main arteries, as the carotid or femoral, we may perceive a slight dull sound, not unlike to that caused by rubbing one finger rather briskly against another, as in giving a fillip. It is easy to make the sound we hear more distinct, only by pressing the end of the stethoscope a little more firmly upon the part; and now the sound is quite analogous to the bruit de soufflet, heard over the heart when any of its orifices are contracted, or in the hypogastrium, at a certain period of gestation, when the uterus and its contents exercise a compression upon the great arterial trunks in the pelvis. We deem this a much more probable explanation of this latter sound than that given by its discoverer, M. Kergaradec, who attributed it to the passage of the blood into the vessels of the placenta, and hence denominated it the "souffle placentaire."

As to the cause of the arterial bruit, we think that there can be only one opinion, viz. that it proceeds from the rapid friction and shock of the blood upon the surface of the arterial tube; the term, therefore, "bruit de frottement" which we (M. B.) proposed several years ago, would be much more appropriate than the one in use. If, with a syringe, we inject by repeated strokes water into the arteries, and the stethoscope be at the same time applied over a large trunk, we shall hear, at each injection, this "bruit de frottement."

This experiment has been repeatedly performed in the hospital before the students, and they have always easily recognized the sound. M. Pelletan, the professor of medical physics to the Faculty in Paris, states, as the result of his experiments, that when a fluid moves along with a certain velocity, a tube, whose inner surface is quite smooth, no sound or murmur can be heard; (M. Bouillaud does not quite agree with him here); but that it is immediately appreciable, if the surface presents any roughnesses or inequalities; and, moreover, that a certain degree of vibratory motion is then felt in the tube; the motion also being much more considerable, if there be any obstacle to the free stream within. It is now very generally admitted by the profession, that the theory proposed by Laennec to explain the bruit de soufflet is quite erroneous.

This illustrious author, prefacing his description by the remark, that it must depend upon either a "particular vital condition, a sort of spasm or tension of the artery, or on a particular state of the blood, or on the manner in which it is moved along," at length gives the preference to the first of these hypotheses, and attempts to confirm and illustrate it, by telling us that the bruit of the arteries, and that of muscles in action, are identically the same; he utterly puts out of consideration the agency of any mechanical obstacle from organic disease, either in the heart or arteries. In conformity with this doctrine, M. Laennec states that the only deviation from health which, without almost any exception, is co-existent with the bruit de soufflet of the heart and large arteries, is a greater or less intense nervous agitation, and that the degree of this is always proportionate to the extent of the bruit. He says—

"Lorsque le bruit de soufflet existe à la fois dans l'aorte, dans les carotides, et dans les troncs arteriels des membres, le malade est dans un etat d'angoisse et d'anxiété extremes. Si le cœur, et la plupart des arteres, presentent le meme phenomene, la vie est en peril; mais, cependant, il est bien rare que le malade succombe, quand il n'y a pas en meme temps affection organique du cœur." Without denying that certain constitutions of body seem to favour the development of this bruit, and that it is by no means unfrequent in irritable, nervous, hysterical and cachectic patients, even when there is no distinct disease of the heart and arteries, we cannot for one moment entertain the unmeaning idea, that it depends upon "a particular vital state, or a sort of spasm," of the circulatory apparatus. What! is it necessary to plunge into the mysteries of vitalism, to explain the occurrences of the blowing, sawing, and rasping sounds which accompany any contraction of the cardiac orifices? as for us, we still adhere to the opinions which we published in 1823, that the blood, in being propelled through any contracted orifice [the forcing pump remaining of the healthy dimensions], experiences an unusual friction; and this friction produces the bruit, and also that peculiar vibratory motion which Laennec called "fremissement cataire." Still we admit that the bruit de soufflet may, and very often does, exist in the præcordial region, without any co-existent contraction of the heart's orifices; but in this case, be it remembered, the sound is only occasional—it is never constant. Of late years, I have had very frequent opportunities of observing the bruit in arteries, while it could not be heard in the heart itself; and it is of much importance to distinguish between these two sets of cases.

It must seem strange to hospital physicians to be told that in a space of four years Laennec had met with only three or four examples of induration of the valves of the heart in patients who during life had presented the bruit de soufflet and the fremissement cataire. Within the last nine years I may safely aver that not fewer than thirty cases have occurred in my practice, and the correctness of the diagnosis has been uniformly confirmed by the post-mortem examinations. Indeed no organic disease of the heart may be so easily distinguished as contraction of the orifices; and we need not repeat that the bruit heard in such cases is a physical, much rather than a vital effect, or, as Laennec says, "an anomaly of nervous influx." Before we quit the consideration of this great author's opinions, we must add, that it has never occurred to us, as it has to him and some other observers, to discover upon dissection a 'notable' contraction of one or more orifices of the heart, when no 'bruit de soufflet ou de rape' had been heard during life.

M. Bouillaud then proceeds to criticise the opinions of Dr. Corrigan on the supposed new disease, which he thought that he had discovered, viz. "permanent patency of the aortic orifice from disease of the semilunar valves," and most powerfully shews their insufficiency and errors. We have not time to transfer the criticism to our pages, but would strongly advise Dr. Corrigan to peruse it attentively. Upon this part of the subject M. Bouillaud again reminds his readers that in all the experiments which he has performed on fluids moving along dead, as well as along living tubes, he has uniformly found that by merely compressing a part, so as to diminish the calibre of the vessel there, a bruit, altogether similar to the bruit de soufflet, may be produced. That such a pressure or constriction is not however the only and invariable cause of this bruit, is amply certified by its very frequent occurrence in nervous chlorotic females, who are subject to hysterical affections. Of late years a number of young women have been sent by different practitioners to the hospital, for supposed organic disease of the heart. They were subject to violent palpitations of the heart, shortness of breath, and strong and very forcible pulsation of the carotids.

The precordial region did not present dulness on percussion over a greater extent than usual; there was no auscultatory sign of any organic lesion; no blowing, sawing, or rasping noise; and yet these patients could not take the gentlest exercise without suffering from dreadful palpitations and dyspnœa.

In all these patients a peculiar sound of a remarkable intensity was audible over the carotid arteries; in some cases these arteries not only blew and hissed, but cood and even gave out a musical whistle, as Laennec has observed. Between this whistling note and the common bruit de soufflet there are numerous degrees or shades of sound; a very curious one is that which Bouillaud compares to the sound of the little instrument, called by the French "le diable," when struck. In a young chlorotic female it was so remarkable, that, by regulating the degree of pressure on the artery, a sort of gamut of tones, from the lowest, which was a blowing, to the highest, which was a rumbling or snoring sound, was produced; just like the succession or wave of tones on the little instrument we have alluded to. When the arterial bruit arrives at such a degree as in the above case, it is not intermittent or occasional, but is continued; being stronger however during the diastole of the vessel, and acquiring therefore a leaping or "saccadé" character. Its most common seat is in the carotid and subclavian arteries, especially the former; it is heard loudest over the sternal extremity of the clavicle. In the majority of cases this "bruit" or "ronflement de diable" is audible only upon one side; sometimes it might be heard on both, but less distinctly on one than on the other; hitherto M. B. has met with it, much more frequently upon the left than upon the right side. In some cases it ceases for a time and then returns. If the stethoscope be pressed firmly upon the artery, without however interrupting the circulation, the sound becomes louder, and even of a disagreeably grumbling tone—in other cases the sound is very sensibly diminished. The position of the head affects it not a little; in general it is increased if the head be inclined to the opposite side, and the chin be elevated at the same time. What is very interesting and important to remark is, that the sound is much weakened, or it ceases altogether, if the larynx be drawn aside from the artery in question.

If the patient make any effort while we are listening to this bruit de diable, it instantly ceases; just as the sound of a vibrating cord is suddenly arrested by nipping it tightly. The bruit is interrupted also by any compression of the artery nearer to the heart than the point of the applied stethoscope.

By far the greater number of the patients in whom we heard this curious sound were young, delicate, irritable, and chlorotic females; the nutritive and assimilative functions were imperfectly performed, even although the individuals might be fat and apparently stout; there might be an excess of fat; but there was certainly a want of good florid blood. The musical sound occurred, generally in lean emaciated subjects. In all the cases no analogous sound was appreciable in the precordial region: perhaps however the sound of the heart's pulsations was more clear than usual.

The treatment of the patients consisted in the exhibition of quinine and of the preparations of iron; the constitutional health was thereby much improved, but the bruit did not altogether cease, except in one case. It is not easy to account for this curious symptom; and perhaps, before attempting any explanation, it will be wiser to examine it under a variety of aspects; to ascertain, among other things, whether it is confined to the carotid and subclavian arteries, or whether it may sometimes be heard in other large trunks. If the former case be true, then it will be necessary to bear in mind the influence of the adjoining windpipe, even although the sound may, as above stated, sometimes exist in one of the carotids only, and be quite awanting in the other.

We should suppose that the state of the arterial tunics is somehow connected with the production of this musical sound; and as there is an exaltation of it at each diastole of the tube, we are led to infer that the heart at each stroke gives a sort of "coup de fouet" to the vessel, through the medium of its contained fluid. It is important to know that an attentive auscultator cannot possibly confound this "arterial ronflement," or murmur, with the blowing or sawing sounds heard in the precordial region, when any of the orifices of the heart are contracted;—if once distinctly heard, it cannot be mistaken for any other. The musical murmur may be still more easily discriminated. As to the simple and intermittent bruit de soufflet of the arteries, it may exist quite independently of any analogous sound of the heart. In conclusion, says M. Bouillaud, the increase of the friction (which in my opinion is the true cause of the bruit de soufflet, properly so called) may arise from three sources, viz.—1. Any exaltation of the motive power of the heart;—thus we know that the sound is audible in many cases of hypertrophy, and of violent palpitations, even although none of the orifices be at all contracted; 2. A contraction of any point of the canal, through which the blood has to pass; and 3. A jagged, irregular, or in any way roughened state of the surface, along which the column of blood flows. We may add that the density of the moving mass of fluid ought probably to be taken into account; but upon this subject, our ignorance forbids us to say more.

Perhaps some of my brethren may be inclined to adopt the opinions lately so ingeniously proposed and defended by M. Rouanet, as to the cause of the two sounds of the heart, which he attributes to the closing or flapping together of the divisions of the auriculo-ventricular and of the semilunar valves. If they do, they will find no difficulty, upon his theory, of applying all the preceding observations to the correct investigation of the abnormal, cardiac, and arterial sounds.—*Journal Hebdom.*

XIII. ULCERS OF THE OS TINCÆ. The following remarks on, and cases of simple ulceration of the os tincæ, are taken from the work of Mad. Boivin and M. Dugés, to the first volume of which we have adverted at some length in a former number. The present subject is considered in the second volume of that able work.

M. Dupuytren has given some account of simple ulceration of the os tincæ. He observes that exploration with the finger only is not sufficient for the discrimination of the affection, which is readily recognized by means of the speculum. On this head we may refer our readers to a notice of a memoir by M. Ricord, in the last number of this Journal. M. Dupuytren proceeds to observe that the os tincæ being disclosed, the surgeon perceives on one or other lip superficial ulceration of a reddish aspect, confined to the mucous membrane. M. Dupuytren recommends cauterizations.

M. Delpech has observed similar ulcers, and cured them by cauterization with the acid nitrate of mercury, many times applied. M. Marjolin observes that the size of the ulcers varies. Pains in the region of the kidneys, a sensation of weight at the fundament, dragging in the groins, heat in the abdomen and enlargement of it as in hysteria, and frequent flushings of the countenance, with or without leucorrhœa, are, according to the same author, the symptoms usually attendant on the disease. M. Dupuytren has added—pain in coïtus.

Our authors advert to syphilitic ulcerations on the cervix and os tincæ. For an account of these we refer again to our notice of M. Ricord's memoir, although we may observe that much in these cases must depend upon the history and attendant circumstances.

Commencing cancerous ulcerations are often distinguished with extreme difficulty from simple ulcers. Local baths are sometimes successful in curing the complaint, and our authors relate one case in which sarsaparilla was equally effectual. Four cases are detailed.

*Case* 1. Madame M., æt. 42, of sanguineous temperament, had borne seven children, and was recently remarried to a young man. For some months she had experienced a feeling of swelling and weight in the vagina, with discharge of a greenish-white matter.

On examining the parts, M. Dugès found the neck of the uterus low in the vagina and much swollen, but without induration. On employing the speculum this part of the uterus was found to be of a deep red colour, the edges superficially ulcerated and of a reddish-brown hue. The contact of the edges of the instrument with the ulcer had occasioned the loss of about a spoonful of pure blood. The patient lost blood whenever she had connexion.

The event is not related.

*Case* 2. Mad. Cher, wife of a carpenter, æt. 30, of delicate constitution, was unable to suckle her second child on account of milk abscess. In one month after her accouchement the catamenia returned, and flowed regularly for fifteen months. After this the woman complained of pains in the kidneys and in the right groin, and every fifteen days the menses appeared in profusion. A physician prescribed leeches, but without success. About two years after the accouchement our author was consulted.

On examining the parts he found considerable tumefaction of the os tincæ, and extensive, though superficial, ulceration of its anterior lip. He prescribed the frequent application of leeches round the pelvis and to the anus, cupping on the loins, mild aperients, decoction of sarsaparilla, and injections of decoction of poppies with belladonna. Besides these means, the patient was directed to use the warm-bath weekly. In five months the patient was cured; in the following year she became pregnant, and she has had no subsequent relapse.

*Case* 3. M. G. æt. 30, a cook, had a child at the age of twenty-three. The labour was tedious and difficult, and attended with considerable hæmorrhage. Eight days afterwards severe hæmorrhage again occurred. After this she regained her strength, and for six years the catamenial secretion was regular. The menses then became too abundant and too frequent. In April the patient had a great hæmorrhage, followed by the discharge of a yellowish matter in large quantity. In July, she entered the Maison de Santé. The neck of the uterus was found very low in the vagina, the orifice directed backwards and very open, and its edges thickened and fissured by ulceration. On the anterior lip of the os tincæ was a vegetation of the size of a large cherry. M. Dubois proposed excision of the neck of the uterus, but the patient would not consent, and left the institution. At the end of a month she returned, having experienced a violent hæmorrhage in the interval.

She now fell under the care of M. Dumeril, who ordered her the 1-10th of a grain of the deuto-chloruret of mercury in pills, four times daily, with emollient and narcotic injections. The mouth was affected on the sixteenth day, when the medicine was suspended. It was resumed and continued for thirty-five days. On the twentieth day the discharge was so profuse that it seemed as if an abscess within the uterus had given way. The patient quitted the hospital before she was cured, and she has not since been seen. On examination before her departure, the excrescence was found to have disappeared, the neck of the uterus was less developed, and the edges of the os tincæ less hard.

*Case* 4. Mad. de W., æt. 33, a Swiss, experienced, in 1826, a sensation of heat in the parts, and severe lancinating and cutting pains in the bottom of the pelvis. The uterus was rather larger than usual, and slightly painful. The patient, absent from her husband, was subjected to domestic uneasiness, and addicted to masturbation. Under anti-phlogistic treatment, and the discontinuance of her injurious practices, she became much better. In 1829, our author was again called to her. Her health was much affected by the loss of her husband, the catamenia were very irregular, and she laboured under the persuasion that she had cancerous ulceration. On examination, the neck of the uterus was found swollen and exquisitely tender. Leeches to the anus and narcotic injections relieved her. In March, 1830, she was in nearly the same condition as before. The uterus, however, was larger and lower in the pelvis, the anterior border of the os tincæ thicker, softened, excoriated on its surface, and bleeding on the least contact. The unhappy lady had resumed her pernicious practices. After this the patient employed another medical attendant, M. Rullier, and the termination of the case is not detailed.

*Case* 5. Mad. Al., æt. 30, menstruated at the age of 15, married a few months afterwards, was a mother at 16, and, after that, had three natural labours, and three miscarriages, from the third to the sixth month. Each accouchement was followed by considerable loss of blood. This young lady had obstinate constipation, almost constant pains in the loins and inguinal regions, weight about the anus, sense of lassitude in the thighs, and slight leucorrhœa. Slight exercise fatigued, and coïtus was often very painful to her. Examined in January, 1828, the uterus was found increased in length, and the orifice was extremely painful to the touch. By means of the speculum, the os tincæ was found to be about eighteen lines in diameter, of a deep red colour, denuded in parts of its mucous membrane. The exposed surface was of an intense red, contrasting with the lividity of the rest of the cervix.

The treatment employed consisted of leeches to the vulva, astringent injections, followed by narcotic, slightly purgative lavements, dry cupping on various parts of the pelvis, flannel next the skin, and hip-baths. Besides these means absence from the husband was enjoined.

Mad. Al. became much better, and, being allowed conjugal intercourse again, she soon became enceinte. This was in November, 1828, and, in December, she had the usual symptoms preceding abortion. She was bled and kept in bed. In February the same symptoms were renewed, with violent cough. She was again bled, &c. The lady had a long and difficult labour. She recovered after it, but our author understands that her complaint has returned.

Our authors observe that they could narrate several cases of enlargement and ulceration of the cervix uteri, cured by repose, regimen, and antiphlogistic treatment. In the preceding case they deplore the premature return of the patient to connubial intercourse.

### XIV. GRANULAR INFLAMMATION OF THE OS TINCÆ.*

Our authors observe that this affection is rare, very little known, and no where described. It has escaped those who have not employed the speculum.

---

* Mad. Boivin and M. Dugés, Traité des Maladies de l'Uterus, &c.

They divide the affection into two varieties, the subacute and the chronic.

In the former there are pain and deep redness of the os tincæ. The elevations discovered by the speculum are sometimes few in number, as large as peas, firm, and whitish; but more frequently numerous, of the size of millet-seed, whitish, and soft. From their interstices blood flows on the contact of the speculum, in examination with the finger, in coïtus, and in defecation. In the chronic variety the granulations are hard and small, whitish, or red and soft, and without redness or softening of the os tincæ itself.

Our authors are tempted to believe that these granulations are not always of the same character, or arising from the same source. Sometimes they have seen them distinctly depend on a dartrous contamination, (vice dartreux,) and on syphilis. Sometimes they co-exist when chronic, with induration of the cervix or fibrous tumour of the uterus.

The disease is not generally formidable when unaccompanied by serious complications. The treatment must be adapted to the more obvious indications—emollients, with local depletion in the acute stage—more stimulating measures in the chronic—specific treatment when the cause is syphilitic. In the greater number of cases derivatives, caustics, &c. have been productive of much advantage. The great point is to discriminate accurately the affection and its characters.

We will subjoin five of eight cases related by our authors.

*Case* 1. Mad. A. Menet, æt. 35, mother of two children, lived luxuriously till the age of twenty-eight, when reverses of fortune obliged her to give lessons in music, which occasioned much exercise of body and of voice. Our authors observe that uterine affections are common among singers. The menstruation was profuse and irregular, there was leucorrhœa, considerable tumefaction of the uterus, surface of the os tincæ livid and studded with vesicular-like granulations, bleeding from the os tincæ during exploration, defecation, and coïtus; emaciation.

Bleeding from the arm to three palettes, cold injections into the vagina, cupping on the sacrum and perinæum several times repeated, the Enghien mineral waters, and some cups of a bitter decoction daily, were the means adopted. In three months the patient was able to resume her occupations. She was directed to wear flannel, and to abstain from long walks.

*Case* 2. The Countess de C. enjoyed good health till the age of 25, when she met with reverses in fortune. From this time she experienced feelings of weight and pain in the genital organs, with irregular menstruation. In 1824, these symptoms were so distressing, that the patient was obliged to keep her bed. On examination, the os tincæ was found of a reddish brown colour, soft, its anterior lip presenting two small white tumors, each of the size of a pea. This portion of the uterus was very painful, and, on raising the organ with the finger, pain was felt in the left iliac fossa—" a certain index of some morbid adhesion on this side."

Emollient baths—leeches to the pelvis—pills of extract of marigold (souci) and country air, were the means recommended. By the 26th May, the tumours had disappeared, and the patient was better in all respects. She took the baths of Plombieres. Absence from the husband was practised. In 1831, she was quite well. This lady's daughter has a similar affection.

*Case* 3. The Countess de L. æt. 40, had formerly a herpetic affection of the arm and chest, which was injudiciously repelled. Great domestic unhappiness, and legal separation from her husband, were followed by irritation of the genital organs and venereal desires, which lasted sometimes for seven or eight hours together. Nothing had relieved this distressing affection.

On examination, the inside of the labia and entrance of the vagina were observed to be red and dry. The os tincæ was larger and lower than natural, and covered with numerous asperities, giving the finger the sensation of grains of sand. Pushing back the organ occasioned pain in the cervix, and in the inguinal regions. The parietes of the uterus were considerably enlarged. Mad. L. would not permit examination with the speculum. We are not informed of the treatment, nor of the result of this case. It tends to shew the local origin of a nymphomaniacal affection.

*Case* 4. A young woman, æt. 25, died in January, 1824, in consequence of accidental luxation of the two last lumbar vertebræ.

The uterus was of small size, and presented a small fibrous tumour in its anterior wall. Beneath the membrane investing the os tincæ, were soft white granulations, of the size of a pin's head, occupying chiefly the anterior lip. This young woman presented externally all the marks of virginity. Her menstruation had been too copious.

*Case* 5. Mad. L. æt. 45, had been brutally ill-treated by her husband, and had had several miscarriages. She entered the Maison de Santé, with symptoms that we need not stop to describe, and eleven days after her admission, she died of pneumonia.

*Dissection.* Besides effusion into the thoracic cavity, there were about three pounds of yellow serous fluid in the abdomen. The liver was studded with tubercles. There was a tumour of the size of a large orange above the left iliac fossa, another in front of the sacro-iliac synchondrosis on the opposite side. These tumors were cysts in the ovaria, which were much disorganized. There was another cyst on the posterior wall of the uterus. The external surface of the latter organ was covered with small, white, prominent concretions, rounded and hard, like the tissue of the cervix. The cavity of the uterus was occupied by a compact body, irregular, and of whitish-red colour, composed of masses united together by a laminated tissue.

We have introduced this and the preceding article, to lead practitioners to make more particular examinations than they often do, of the condition of the uterus. They may perceive how the symptoms, of which patients usually complain, are common to many local alterations, some of which are perhaps unknown, even by name, to the generality of medical men.

### XV. ACADEMY OF SCIENCES.

*(Continued.)*

*Séances of July.*

### CROUP.

Dr. Maingault read a memoir on the "Urgency of performing Tracheotomy in Croup." The conclusions which he has drawn from his extended researches are the following.

1. That the operation should be performed without delay, when the antiphlogistic and other remedies have failed, or promise little benefit.

2. That the success of the operation is much dependent upon the extent of the inflammation; when it is confined to the larynx, our prognosis may be more favourable.

3. That the opening into the trachea should be made very cautiously, and by repeated strokes with the scalpel, "a plusieurs reprises," because the sudden rushing in of the air through a large aperture may cause asphyxia; and also the ready admission of blood into the windpipe is obviated considerably.

4. That the insufflation of any powder, or the introduction of any liquid, through the wound, into the trachea, ought to be denounced.

### MEDICAL ELECTRICITY.

The attention of the Academy has been called, of late, to the great improvements which M. Molt has introduced into this department of therapeutics. MM. Alibert, Dubois, and Desgenettes were appointed to inspect his establishment, and they have made a most favorable report. The following is a list of the electrical instruments which are proved to be the most useful.

1. The electric brush, which imparts an electric current of great activity, without either spark or shocks.

2. Electric sounds or bougies; for the purpose of being introduced into the urethra and vagina, in paralytic affections of the bladder—in amenorrhœa, &c.

3. A compressing pump, to inject electrified water. The electric fluid is conveyed by means of a current of common or of mineral water; its advantages have been experienced in amenorrhœa.

4. An electrical projector, to communicate the electric fluid under the form of a crackling cool breeze. Cases of amaurosis, and some forms of neuralgia, have been benefited by the use of this instrument.

### Embryology.

Dr. Coste, the associate of the late distinguished M. Delpech, has been long engaged in researches respecting the development of the fœtus; the scope and character of these may be drawn from the following brief detail. From numerous minute examinations of the ovaries of mammiferous animals, he has come to the same conclusion as Baer did, viz. that the vesicles of Graaf are, in all respects, similar to the ova of birds. These vesicles are composed of an inorganic external vitelline membrane; and within this there is a transparent liquid, holding small granular particles in suspension.

This liquid is to be viewed as the proper vitellus; for it is at its expense that the cicatricula begins to be developed. On the inner surface of the vitelline membrane, we find a small circular lamina, free and moveable, resting on the vitellus; this lamina is quite analogous to the cicatricula, in the ova of birds. At the time of conception, the Graafian vesicle is detached from the ovary; it then enters, and passes along the fallopian tube, till it reaches the cavity of the womb. The cicatricula, whose earliest development commences while the vesicle is in the tube, gradually extends itself in all directions, till at length it occupies almost the whole inner surface of the vitelline membrane; it then begins to close itself, as a sac, towards that point which is diametrically opposite to the embryonic germ. As the germ increases in size, it absorbs all the contents of the ovum or vesicle; and it now, therefore, begins to be attached to the uterus. The point by which it is attached, is that which we have mentioned above as being opposite to the germ.

### Helminthology.

The histories of several cases of the larvæ of the æstrus, or gad-fly, having been found in the human subject, were presented to the Academy, and submitted to the review of M. Geoffroy St. Hilaire. In the first case, observed by M. Roulin at Maraquita, in Colombia, there was a tumour of the size of a large chesnut on the scrotum; the apex was very red, and presented a small opening; this having been enlarged by an incision, gave issue to a whitish pyriform larva, about 10 lines long, and five or six broad at the largest part. The author states that this larva was quite similar to those very frequently found under the skin of the cattle of the country. In a jaguar which he killed on the Cordilleras, he found a great number of living larvæ of the æstrus, under the skin of the flanks. In the case detailed by M. Guérin, the larvæ were contained in the pustules scattered over the body of a negro, who lay sick of variola at the island of Martinique; the length of these larvæ was about 15 millimetres, and the breadth about two. They were of a white colour, indistinctly articulated, and provided with a mouth and anus, easily recognizable with a magnifying glass. M. St. Hilaire very properly says, that it is much to be regretted, that in none of the instances were the larvæ watched till the period of their tranformation; but perhaps we cannot well expect so great a degree of resolution in a patient, as voluntarily to submit to procrastinated pain, which he knows may be easily and effectually got rid of, the extraction of the larvæ being abundantly simple.

### XVI. ACADEMY OF MEDICINE.

*(Continued.)*

*Séances in June.*

ABNORMAL CAVITY IN THE HEART.

M. Cullerier exhibited the heart of a woman, who had died of phthisis, in which there was a cavity in the inter-ventricular septum.

No symptoms during life had led the physician to anticipate any abnormal state of the organs.

TRAUMATIC TETANUS.

M. Lepelletier communicated the details of an interesting case of fatal tetanus supervening on amputation of the leg; the patient died on the eighth day after the operation. On dissection the sciatic nerve was found vehemently red and injected with blood, from the wound up as far as the pelvis.

M. L. regards this inflammation of the neurilema as the exciting cause and local origin of the tetanus.

*Public Séance of the 9th July.*

The Academy met in their great hall to announce the prize-subjects for the years 1834 and 1835, and to hear three orations by M.M. Marc, Reveillé-Parise, and Pariset.

The President of the Academy, M. Marc, treated of monomania in its relations with legal medicine and jurisprudence. He forcibly illustrated the existence and characters of this Protean mental malady, and in opposition to many learned lawyers and magistrates, endeavoured to prove that not a few transgressions and crimes are the result of a morbid state of the body, and are not in truth the offspring of a mind capable of deliberation.

The second oration was upon that very just saying of Aristotle, "that most men of genius are affected with melancholy." M. R. ingeniously attempted to shew that the very existence of genius is attributable to an exalted activity and susceptibility of the nervous system, whether this be original or acquired; and that at the same time the very excess of these functions disposes individuals to the disappointment and mental chagrin, which in truth constitutes melancholy.

The concluding oration was delivered by the Secretary, and was well worthy of the noble theme, an eulogium on the late Baron Cuvier.

CHOLERA MORBUS.

M. Kerandren read a letter from M. Guibert, the surgeon of the frigate Melpomene, which has been recently on the Lisbon station. This ship was lying in the Tagus for three months, with a fine healthy crew, exempt from cholera, although the disease was rife on shore. On the 30th June, the pestilence broke out on board most unexpectedly and with great violence. Many of the sailors died in the course of a few hours. The sick were sent to the hospital on shore (an imprudent step) and the ship went to sea on the 3d of July, but the disease travelled as quickly as she did, and for three or four days attacked a number of the crew. In most of the severe cases there were no premonitory symptoms: the poor fellows being as it were stricken at once with death.

DIPTHERITE, OR ANGINA MEMBRANACEA.

M. Gendron regards diptherite and croup as the same disease, only occupying different parts; in the one case the cavity of the mouth and fauces are chiefly affected, in the other, the larynx and trachea. In both the character of the inflammation is the same, and there is the same disposition to the deposit of the false membranes. The first stage of the diptherite demands active depletions; in the second stage, however, when well-formed concretions begin to appear in the throat, our hopes of success must rest chiefly on the application of caustics to the diseased surface. The author prefers for this purpose the nitrate of silver, which may be used either in the solid or fluid form. Some practitioners recommend highly the use of the mineral acids, especially the muriatic.

